# *HoxBlinc* lncRNA reprograms CTCF-independent TADs to drive leukemic transcription and HSC dysregulation in NUP98-rearranged leukemia

**DOI:** 10.1172/JCI184743

**Published:** 2025-01-30

**Authors:** Karina Hamamoto, Ganqian Zhu, Qian Lai, Julia Lesperance, Huacheng Luo, Ying Li, Nupur Nigam, Arati Sharma, Feng-Chun Yang, David Claxton, Yi Qiu, Peter D. Aplan, Mingjiang Xu, Suming Huang

**Affiliations:** 1 Division of Pediatric Hematology/Oncology, Department of Pediatrics, Pennsylvania State University College of Medicine, Hershey, Pennsylvania, USA.; 2 Department of Molecular Medicine, University of Texas Health Science Center at San Antonio, San Antonio, Texas, USA.; 3 Genetics Branch, Center for Cancer Research, National Cancer Institute (NCI), NIH, Bethesda, Maryland, USA.; 4 Department of Pharmacology, Pennsylvania State University College of Medicine, Hershey, Pennsylvania, USA.; 5 Department of Cell Systems and Anatomy, University of Texas Health Science Center at San Antonio, San Antonio, Texas, USA.; 6 Division of Hematology/Oncology, Department of Medicine, Pennsylvania State University College of Medicine, Hershey, Pennsylvania, USA.; 7 Penn State Cancer Institute, Hershey, Pennsylvania, USA.; 8 Department of Cellular and Molecular Physiology, Pennsylvania State University College of Medicine, Hershey, Pennsylvania, USA.

**Keywords:** Genetics, Hematology, Epigenetics, Hematopoietic stem cells, Oncogenes

## Abstract

Although nucleoporin 98 (NUP98) fusion oncogenes often drive aggressive pediatric leukemia by altering chromatin structure and expression of homeobox (*HOX*) genes, underlying mechanisms remain elusive. Here, we report that the Hoxb-associated lncRNA *HoxBlinc* was aberrantly activated in NUP98-PHF23 fusion–driven leukemias. *HoxBlinc* chromatin occupancies led to elevated mixed-lineage leukemia 1 (MLL1) recruitment and aberrant homeotic topologically associated domains (TADs) that enhanced chromatin accessibilities and activated homeotic/hematopoietic oncogenes. *HoxBlinc* depletion in NUP98 fusion–driven leukemia impaired *HoxBlinc* binding, TAD integrity, MLL1 recruitment, and the MLL1-driven chromatin signature within *HoxBlinc*-defined TADs in a CCCTC-binding factor–independent (CTCF-independent) manner, leading to inhibited homeotic/leukemic oncogenes that mitigated NUP98 fusion–driven leukemogenesis in xenografted mouse models. Mechanistically, *HoxBlinc* overexpression in the mouse hematopoietic compartment induced leukemias resembling those in *NUP98-PHF23*–knockin (KI) mice via enhancement of *HoxBlinc* chromatin binding, TAD formation, and Hox gene aberration, leading to expansion of hematopoietic stem and progenitor cell and myeloid/lymphoid cell subpopulations. Thus, our studies reveal a CTCF-independent role of *HoxBlinc* in leukemic TAD organization and oncogene-regulatory networks.

## Abstract 2

*HoxBlinc* serves as downstream oncogenic regulator that controls specificity of NUP98 fusion-associated TAD, chromatin accessibility and Hox/HSC gene transcription critical for HS/PC perturbation and leukemogenesis.

## Graphical abstract

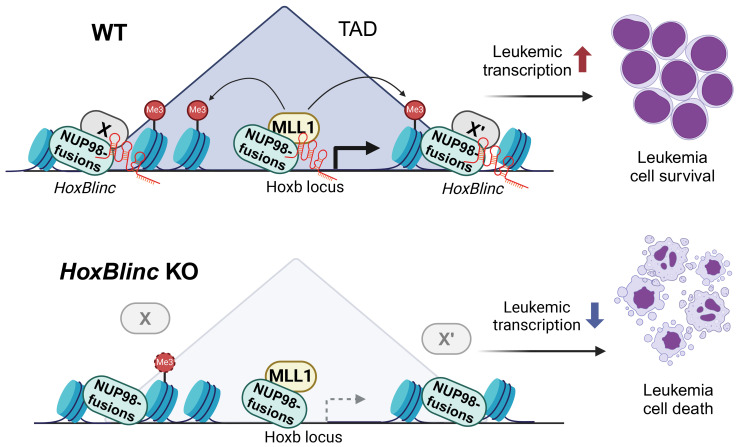

## Introduction

Nucleoporin 98 (*NUP98*) gene is often fused with a variety of partners in hematopoietic malignancies, especially in aggressive pediatric leukemias with adverse patient outcomes (1–6). The *NUP98* gene encodes a nucleoporin protein that serves as a structural component of the nuclear pore complexes (NPCs) involved in the transport of macromolecules between the nucleus and cytoplasm. Recent discoveries have revealed that the NPC has multiple cellular functions besides mediating the molecular exchange between the nucleus and cytoplasm (7, 8). It was reported that NUP98 can bind to chromatin to regulate gene expression, in part by recruiting histone H3 methyltransferase complex of proteins associated with SET1 (COMPASS) complexes (9, 10), suggesting an additional function in epigenetic and transcriptional regulation (11). In NUP98 translocation–driven leukemias, the N-terminal region of NUP98 is fused to 1 C-terminal region of more than 30 fusion partners. These fusion partners are often homeobox transcription factors (TFs), such as homeobox A9 (HOXA9), homeobox D13 (HOXD13), plant homeodomain (PHD) finger 23 gene (PHF23), or hematopoietically expressed homeobox (HHEX), and components of epigenetic machinery, for instance lysine-specific demethylase 5A (KDM5A), nuclear receptor-binding SET domain protein 1 (NSD1), or bromodomain PHD finger transcription factor (BPTF) (12). Oncogenic NUP98 fusions, especially with those fusion partner genes containing the homeobox domain, result in aberrant activation of HOX genes, especially HOXA cluster of oncogenes, thereby altering hematopoietic stem and progenitor cell (HSPC) differentiation and self-renewal leading to leukemic transformation (6, 12). Mechanistically, the N-terminal region of NUP98 possesses an intrinsically disordered region (IDR) enriched in a phenylalanine-glycine motif that promotes NUP98-HOXA9 phase separation in the nucleus and drives active topologically-associated domain (TAD) organization to facilitate oncogenic transcription activity and HSPC transformation (13–15). However, it remains elusive how global genome reorganization leads to specific alternations of gene-regulatory networks affecting characteristic homeotic/hematopoietic oncogenic pathways.

*NUP98*-*PHD23* fusion results from a cryptic translocation t(11;17) (p15;p13) creating a fusion oncoprotein that juxtaposes the N-terminal region of NUP98 to the C-terminal portion of PHF23 (16). NUP98-PHF23 fusion occupies chromatin by recognizing H3K4me3 marks in specific regions of the genome such as *HOXA*, *HOXB*, and *MEIS1* loci to aberrantly activate these homeotic oncogenes (17, 18). It is particularly interesting that, although NUP98-PHF23 drives AML in humans, the *NUP98-PHF23*–KI mouse models develop diverse leukemias including myeloid, erythroid, T cell, and B cell leukemias (17). It was suggested that NUP98-PHF23 mediated persistent upregulation of homeotic oncogenes contributed to the leukemogenesis seen in KI mouse models via preventing hematopoietic differentiation and promoting self-renewal of hematopoietic progenitors (17). Despite the critical role of NUP98-PHF23 in HOXA/B gene activation and leukemogenesis, it remains poorly understood whether and how NUP98-PHF23 or other NUP98 fusions recognize the *HOXA/B* gene regions, resulting in homeobox oncogene aberration and dysregulation of multiple hematopoietic lineages leading to leukemogenesis.

Dysregulation of HOXA/B genes is a dominant mechanism of HSPC alteration and leukemic transformation (19). Several HOXA/B-associated long noncoding RNAs (lncRNAs), such as *HOTTIP* and *HOXBLINC*, are known to control HOX locus chromatin structure and gene expression (20). *HoxBlinc* lncRNA is transcribed from anterior Hoxb loci in early hemopoiesis and regulates anterior/middle Hoxb gene expression to specify early hematopoietic progenitors/precursors. It was further revealed that *HoxBlinc* recruits SET domain containing protein 1A (SETD1A) mixed-lineage leukemia 1 (MLL1) complexes to Hoxb loci and controls gene expression (21). Interestingly, *HOXBLINC* lncRNA is aberrantly activated in NPM1^C+^-mutant–driven acute myeloid leukemia (AML) to facilitate signature HOXA/B chromatin structure and gene expression (22). In the current studies, we demonstrated that *HoxBlinc* lncRNA was ectopically activated in NUP98-PHF23– and NUP98-HOXA9–driven leukemias and acted as a critical downstream regulator of the NUP98-driven chromatin structure and transcription signature. *HoxBlinc* lncRNA mediates and reprograms NUP98 fusion oncoprotein–driven Hoxa/b TAD formation, chromatin structure, and gene expression changes in a CCCTC-binding factor–independent (CTCF-independent) manner. Mechanistically, we demonstrated that overexpression of *HoxBlinc* lncRNA in the mouse hematopoietic compartment (*HoxBlinc*-Tg) led to signature TAD reorganization, chromatin structure changes, and homeotic oncogene activation resembling the phenotype driven by NUP98 fusions. Functionally, *HoxBlinc*-Tg perturbed HSPC activity and skewed normal hemopoiesis toward myeloid and/or lymphoid lineages, leading to the development of diverse leukemias in mice similar to those that developed in *NUP98*-*PHF23* KI mice. Thus, our results revealed that *HoxBlinc* lncRNA serves as a downstream oncogenic regulator that controls the specificity of NUP98 fusion–associated 3D genome organization, chromatin accessibility, and Hox/hematopoietic gene transcription critical for HSPC perturbation and leukemogenesis.

## Results

### HoxBlinc lncRNA is highly activated and binds to Hoxa/b domains in NUP98-PHF23–driven leukemia.

**Figure 1:**
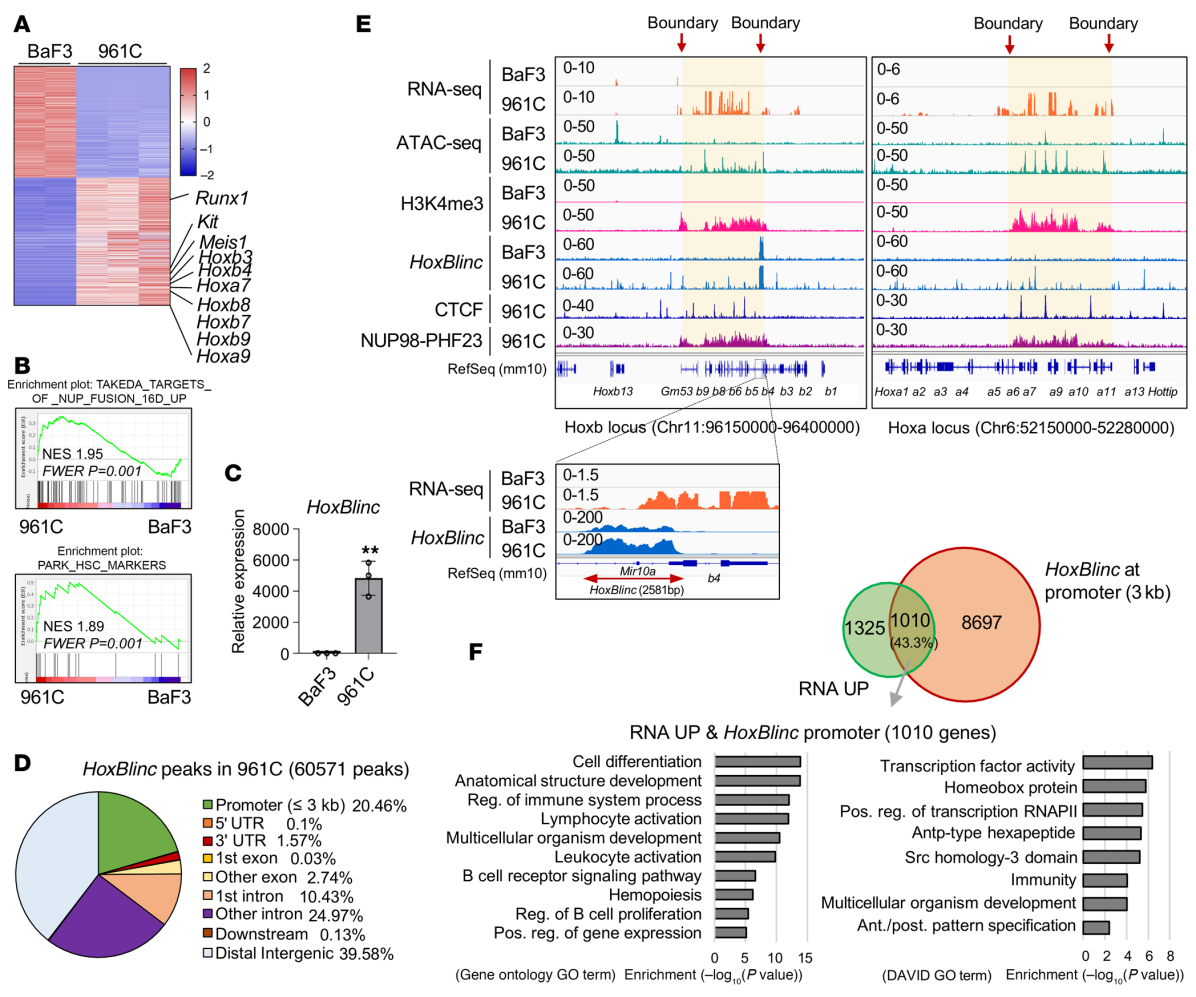
*HoxBlinc* lncRNA is highly activated and binds to Hoxa/b in NUP98-PHF23–driven leukemia. (**A**) Heatmap of the *z* score in normalized differentially expressed genes (DEGs) by DEseq2 in RNA-Seq (961C vs. BaF3). DEGs were selected according to a log_2_(fold change) of greater than 1 and an FDR of less than 0.05. (**B**) GSEA of DEGs using the TAKEDA_TARGETS_OF_NUP98_HOXA9_FUSION_ 16D_UP (M15588) gene set and the PARK_HSC_MARKERS (M6509) gene set. (**C**) Mouse *HoxBlinc* expression levels in BaF3 and 961C cells were determined by RT-qPCR. Data are presented as the mean ± SD from three independent experiments. **P ≤ 0.01, by 2-tailed, unpaired Student’s *t* test. (**D**) Pie chart of global *HoxBlinc* ChIRP-Seq distribution in 961C B-ALL cells carrying the NUP98-PHF23 fusion (60,571 peaks). (**E**) RNA-Seq, ATAC-Seq, H3K4me3 CUT&RUN, *HoxBlinc* ChIRP-Seq, and CTCF/NUP98-PHF23-V5 ChIP-Seq analysis of changes in RNA levels, chromatin accessibility, H3K4me3 modification levels, *HoxBlinc* lncRNA, and CTCF and NUP98-PHF23 bindings at *Hoxa* and *Hoxb* loci in BaF3 versus *NUP98-PHF23*–KI 961C cells. The *HoxBlinc*-transcribed region is enlarged. *HoxBlinc-*defined active TADs/sub-TADs at *Hoxa/b* loci are highlighted in yellow. (**F**) Top: Overlapping of RNA-upregulated genes in 961C cells with *HoxBlinc* binding at the promoter (within 3 kb). Bottom left: GO analysis of 1,010 overlapping genes. Bottom right: Database for Annotation, Visualization, and Integrated Discovery (DAVID) GO functional annotation enrichment analysis of 1,010 overlapping genes. Ant./post., anterior/posterior; Pos. reg., positive regulation.

Leukemic cells from *NUP98-PHF23*–KI mice exhibited abnormal upregulation of Hoxa/b and homeotic oncogenes enriched for a stem cell–like gene expression signature (17) that is reminiscent of the gene expression signature in *NPM1^C+^* AML cells (22). Given that the Hoxb-associated lncRNA *HoxBlinc* is activated in *NPM1^C+^* AML cells to promote *NPM1^C+^*-driven leukemogenic *Hoxa/b* clusters and the *Meis1* signature (22), we sought to test whether and how *HoxBlinc* lncRNA is a critical downstream mediator of the NUP98-PHF23 fusion oncoprotein to drive the homeotic leukemic transcription signature and leukemogenesis. 961C is a primary B cell acute lymphoblastic leukemia (B-ALL) cell clone derived from NUP98-PHF23–derived B-ALL mouse models. RNA-Seq revealed that 961C cells exhibited HOX signature gene expression patterns similar to those observed in *NUP98-PHF23*–KI mouse bone marrow (BM) cells (Figure 1A) (17). Gene ontology (GO) analysis of RNA-Seq datasets comparing 961C cells with control BaF3 B cell precursors revealed that the upregulated genes in 961C primary B-ALL cells are those involved in lymphocyte/mononuclear cell proliferation, immune system processes, hemopoiesis, B cell activation and proliferation, transcriptional regulation, and anterior/posterior pattern specification (Supplemental Figure 1A). Gene set enrichment analysis (GSEA) further revealed that genes altered by *NUP98-PHF23* KI were enriched in NUP98-HOXA9 and hematopoietic stem cell (HSC) gene signatures (Figure 1B). Interestingly, we observed that *HoxBlinc* lncRNA, which was previously found to play an important role in early hematopoietic progenitor specification (21), was ectopically activated (Figure 1, C and E bottom).

*HOXBLINC* has been shown to regulate *HOXB* gene expression in early hemopoiesis and *NPM1^C+^* AML by recruiting MLL1-SETD1A complexes (21, 22). To further determine whether *HOXBLINC* mediates and regulates NUP98-PHF23 epigenetic landscape alteration and signature homeotic oncogenic transcription, we examined genome-wide *HoxBlinc* chromatin binding by chromatin isolation using RNA purification in conjunction with next-generation sequencing (chromatin isolation by RNA purification [ChIRP-Seq]) as well as corresponding H3K4me3 enrichment, CTCF binding, NUP98-PHF23 binding, and chromatin accessibility by CUT&RUN, ChIP-Seq, and assay for transposase-accessible chromatin using sequencing (ATAC-Seq) in NUP98-PHF23–transformed 961C B-ALL cells versus control BaF3 B cell precursors. Compared with the *LacZ* control, biotinylated *HoxBlinc* probes specifically enriched *HoxBlinc* RNA, which bound strongly to anterior *Hoxb4/5* genes, but weakly to the posterior *Hoxb9* gene (Figure 1E, Supplemental Figure 1B). ChIRP-Seq revealed that *HoxBlinc* RNA mainly occupied promoter regions (20.46%), intergenic regions (39.58%), and intron regions (35.4%), suggesting its roles in transcriptional regulation (Figure 1D). *HoxBlinc* bound to Hoxb loci specifically in domain-encompassed *Hoxb4*, *b5*, *b6*, *b8*, *b9*, and *Gm53* genes, as well as in Hoxa domain–encompassed *Hoxa6*, *a7*, *a9*, *a10*, and *a11* genes, where *HoxBlinc* also occupied the boundaries of both active Hoxb and Hoxa domains (Figure 1E, yellow highlights). As a control, we noted that CTCF did not co-occupy at the chromatin boundaries of active Hoxa/b domains driven by NUP98 fusion (Figure 1E). Interestingly, we observed that the H3K4me3 and ATAC peaks were aberrantly enriched in the *HoxBlinc*-defined active Hoxa/b domains, leading to transcriptional activation of genes residing within the active chromatin domains in 961C cells as compared with BaF3 control cells (Figure 1E). Integrated analysis of the ChIRP-Seq and RNA-Seq datasets revealed that 43.3% of genes upregulated in 961C B-ALL cells by RNA-Seq were occupied by *HoxBlinc* lncRNA at promoters (Figure 1F, top) and involved in pathways regulating transcription regulation and homeobox protein function (Figure 1F, bottom right). GO analysis showed that *HoxBlinc* binding–related upregulated genes are involved in cell differentiation, lymphocyte activation, immune response, B cell receptor activation/B cell proliferation, regulation of gene expression, and hemopoiesis (Figure 1F, bottom left). Furthermore, among the 2,335 upregulated genes in 961C cells, 565 genes (24.2%) exhibited an increase in chromatin accessibility by ATAC-Seq (Supplemental Figure 1C). GO term analysis of these overlapping genes revealed that they are consistently involved in multicellular organismal processes, anatomical structure development, cell differentiation, hemopoiesis, and positive regulation of transcription (Supplemental Figure 1D). Thus, *HoxBlinc* may define the active Hoxa/b domain boundaries and thereby regulate chromatin structural and transcriptional changes in NUP98-PHF23–driven leukemia.

### HoxBlinc defines chromatin boundaries of Hoxa/b TADs in NUP98-PHF23–driven leukemia.

**Figure 2:**
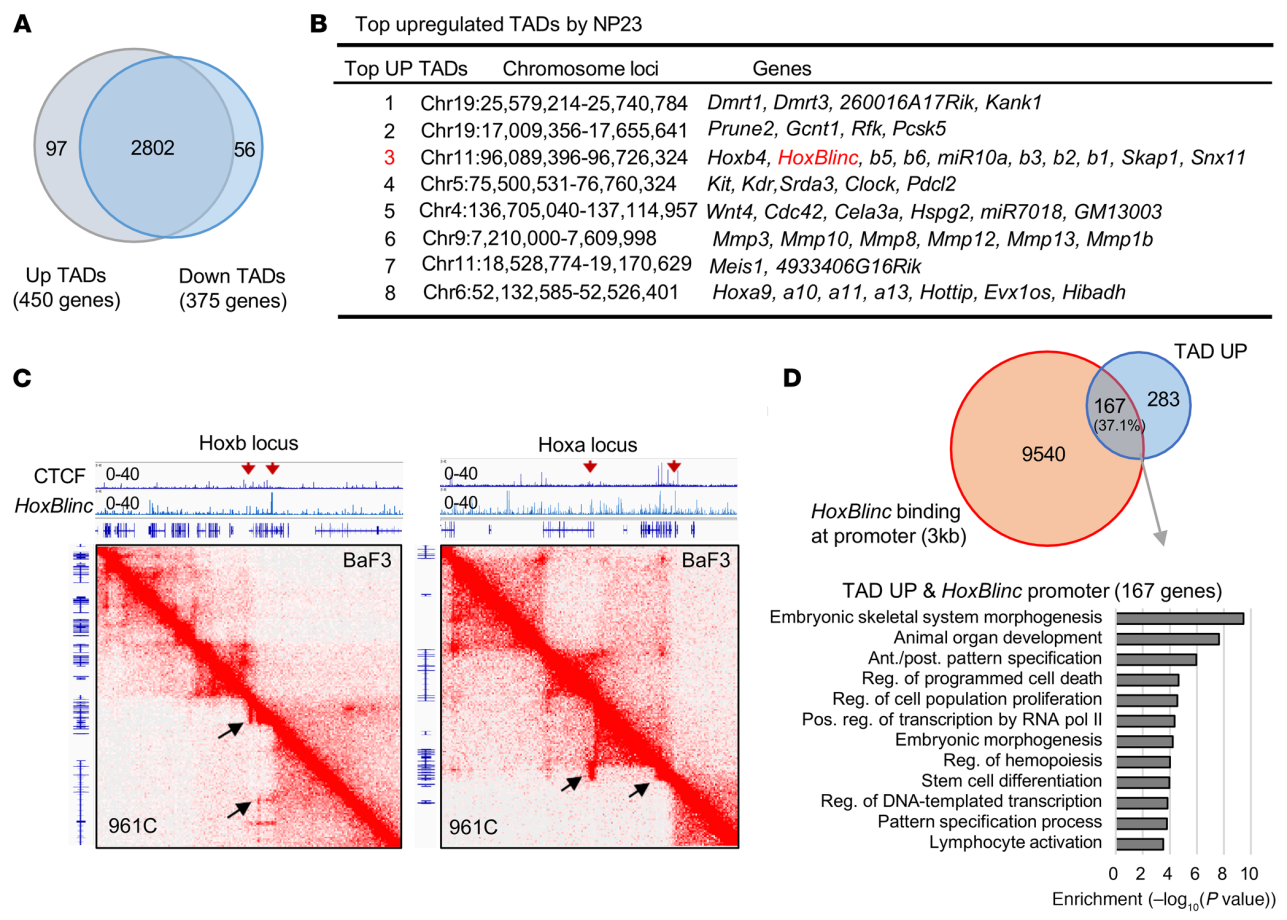
*HoxBlinc* defines chromatin boundaries of Hoxa/b TADs in NUP98-PHF23–driven leukemia. (**A**) Pie chart shows overlapping of TADs by Hi-C in 961C B-ALL cells compared with BaF3 B cell precursors. The domain score for an altered TAD was normalized (quantile normalization) by subtracting the mean of all TAD Hi-C signals. ANOVA was used to identify significantly altered TADs (Bonferroni-corrected *P* ≤ 0.05). (**B**) Top 10 upregulated TADs in 961C B-ALL cells as compared with BaF3 B cell precursors. (**C**) HiC-Seq–interacting maps in part of the mouse chromosome 6 and chromosome 11 regions of *Hoxa* and *Hoxb* loci comparing BaF3 B cell precursors and 961C B- ALL cells. Black arrows indicate upregulated TADs/sub-TADs in 961C cells. Top panel shows *HoxBlinc* and CTCF binding in the corresponding Hoxa/b loci. (**D**) Top: Pie chart shows overlapping of TAD-upregulated (TAD UP) genes and promoters bound by *HoxBlinc* lncRNA. Bottom: GO analysis of these 167 overlapping genes.

To further explore whether *HoxBlinc* lncRNA indeed defines the chromatin boundaries of Hoxa/b TADs, we performed Hi-C analysis of NUP98-PHF23–transformed B-ALL 961C cells versus control BaF3 B cell precursors. Hi-C analysis revealed that NUP98-PHF23–transformed 961C cells had an increase of 97 TADs and a decrease of 56 TADs (Figure 2A). The increased TADs encompassed 450 genes, of which 131 genes (29.1%) showed aberrant activation of gene transcription by RNA-Seq (Supplemental Figure 2A, left). These upregulated TAD-encompassed active genes were primarily involved in anterior/posterior patterning, multicellular organismal processes, transcription, cell differentiation, B cell activation, and hemopoiesis (Supplemental Figure 2A, right). Notably, the top 10 upregulated TADs encompassed *Hoxb*, *Hoxa*, *Kit*, and *Meis1* loci that play an important role in HSPC function and leukemogenesis (Figure 2, B and C, and Supplemental Figure 2, B and C). Interestingly, the boundaries of these increased TADs were occupied by *HoxBlinc* lncRNA, but not CTCF (Figure 2, B and C and Supplemental Figure 2, B and C). When we compared global *HoxBlinc* binding and CTCF binding in the 961C cell genome, only a very small fraction (1,337 peaks, < 1%) of *HoxBlinc*-binding sites were co-occupied with CTCF. Moreover, the majority of *HoxBlinc*- or CTCF-binding sites did not overlap with each other (Supplemental Figure 2D). Notably, 37.1% of upregulated genes encompassed by the upregulated TADs in 961C cells were occupied by *HoxBlinc* lncRNA in promoter regions (Figure 2D, top). These genes were involved in embryonic skeleton morphogenesis, anterior/posterior patterning, cell population proliferation, transcription regulation, stem cell differentiation, and hemopoiesis (Figure 2D, bottom). Together, our data suggested that *HoxBlinc* lncRNA plays an important role in oncogenic homeotic TAD formation, chromatin remodeling, and leukemic signature gene transcription, perhaps by acting downstream of the NUP98-PHF23 fusion oncoprotein.

### HoxBlinc is essential for NUP98-PHF23–driven homeotic oncogene expression and leukemogenesis.

**Figure 3:**
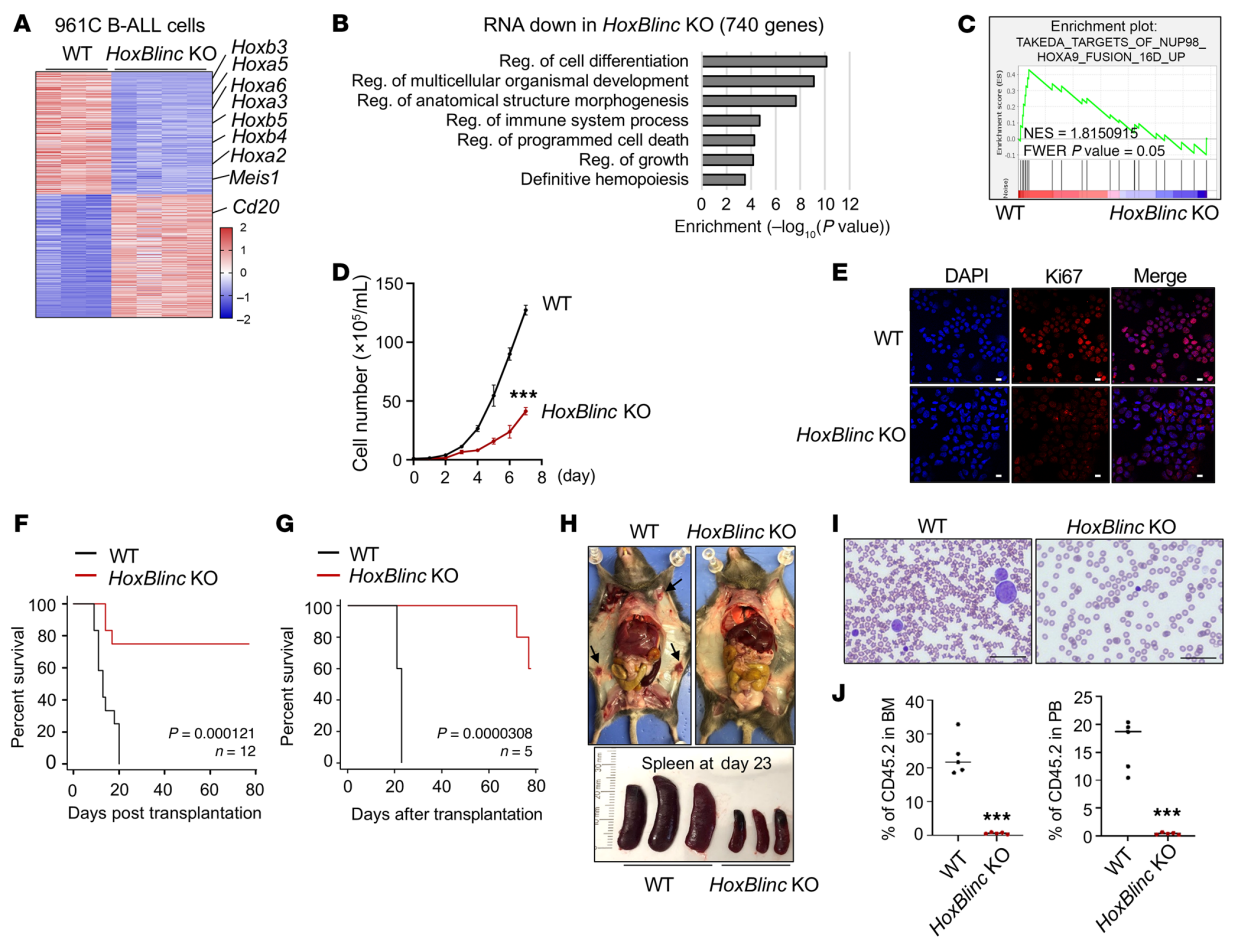
*HoxBlinc* is essential for the NUP98-PHF23–driven leukemic homeotic gene signature and leukemogenesis. (**A**) Heatmap of normalized DEGs by DEseq2 in RNA-Seq of WT versus *HoxBlinc-*KO 961C cells. DEGs were selected using log_2_(fold change) of greater than 0.58 and an FDR of less than 0.05. (**B**) GO analysis of 740 downregulated genes upon *HoxBlinc* KO in 961C B-ALL cells. (**C**) GSEA of DEGs between WT and *HoxBlinc-*KO in 961C cells using the TAKEDA_TARGETS_OF_NUP98_HOXA9_FUSION_ 16D_UP (M15588) gene set. NES, normalized enrichment score; FWER, family-wise error rate. (**D**) Cell proliferation curve of 961C and *HoxBlinc-*KO 961C cells over a 7-day period. Data are presented as the mean ± SD from 3 independent experiments. ***P ≤ 0.001, by 2-tailed, unpaired Student’s *t* test at day 7. (**E**) Ki67 immunofluorescence images comparing WT and *HoxBlinc-*KO 961C cells. Scale bar 10μm. (**F**) Kaplan-Maier survival curve of mice transplanted with 961C and *HoxBlinc-*KO 961C cells. *n* = 12/group. *P* = 0.000121, by log-rank test. (**G**) Kaplan-Maier survival curve for mice transplanted with A1929 and *HoxBlinc-*KO A1929 primary B-ALL cells. *n* = 5/group. *P* = 0.0000308 by log-rank test. (**H**) Whole body and spleen images of A1929 cell– or *HoxBlinc-*KO A1929 cell–transplanted mice at day 23 after transplantation. (**I**) MGG-stained images of PB smears from WT 961C cell– with *HoxBlinc-*KO 961C cell–transplanted mice at post-translation day 23. Scale bar 50μm. (**J**) FACS analysis of CD45.2^+^ cell population in BM and PB from recipient mice 23 days after transplantation of WT 961C or *HoxBlinc-*KO 961C cells. *n* = 5. Data are presented as the mean ± SD. ***P ≤ 0.001, by 2-tailed, unpaired Student’s *t* test.

To validate whether *HoxBlinc* lncRNA is required for the NUP98-PHF23 fusion oncoprotein–driven homeotic gene signature and leukemogenesis, we knocked out the promoter region of *HoxBlinc* lncRNA using sgRNA-targeted CRISPR editing in two NUP98-PHF23–transformed B-ALL primary cells, 961C and A1929, and confirmed this KO by Sanger sequencing (Supplemental Figure 3, A and B). We found that KO of *HoxBlinc* largely diminished *HoxBlinc* transcripts (Supplemental Figure 3C). Given that *HoxBlinc* lncRNA acts as a potential regulator of Hox gene transcription in normal and malignant hemopoiesis (21, 22), we performed RNA-Seq to compare changes in NUP98-PHF23–driven transcriptome profiles in WT versus *HoxBlinc-*deficient 961C or A1929 B-ALL cells. KO of *HoxBlinc* resulted in a significant (FDR-corrected *P* < 0.05) decrease in Hox and homeotic oncogene transcription, including of *Hoxa*, *Hoxb*, *Meis1* genes, as measured by RNA-Seq (Figure 3A and Supplemental Figure 3D). The downregulated genes were those primarily involved in regulation of cell differentiation, immune system processes, apoptosis, and definitive hemopoiesis (Figure 3B and Supplemental Figure 3E). GSEA indicated that NUP98-HOXA9 fusion signature genes and pathways were impaired and highly enriched in downregulated genes by *HoxBlinc* KO (Figure 3C), suggesting that activation of *HoxBlinc* lncRNA was a key event for NUP98 fusion–driven oncogenic *Hox* gene expression.

Next, we sought to determine whether *HoxBlinc* lncRNA regulates NUP98 fusion–driven leukemic cell proliferation in vitro and leukemogenesis in vivo. KO of *HoxBlinc* in 961C or A1929 B-ALL cells carrying NUP98-PHF23 fusion led to significantly inhibited cell proliferation, as determined by cell viability counts and Ki67 staining assays (Figure 3, D and E, and Supplemental Figure 3F). To further test whether *HoxBlinc* loss affects NUP98-PHF23–driven leukemogenesis, we transplanted 1 × 10^6^ WT or *HoxBlinc*-KO cells into busulfan-treated CD45.1 recipient mice. All mice transplanted with WT 961C or A1929 cells died within 16 or 23 days, respectively (Figure 3, F and G). The mice transplanted with WT cells exhibited splenomegaly and enlarged lymph nodes (Figure 3H) with increased immature leukemic blasts in the peripheral blood (PB) (Figure 3I and Supplemental Figure 3G). In contrast, KO of *HoxBlinc* in both primary B-ALL cells dramatically prolonged survival of the transplanted mice, with half of them surviving more than 80 days after transplantation (Figures 3, F and G). Mice that received *HoxBlinc*-KO cells had a relatively normal spleen size without enlarged lymph nodes at the time when the animals receiving control B-ALL cells died (Figure 3H). FACS analysis revealed that *HoxBlinc* KO dramatically decreased donor CD45.2^+^ and CD19^+^/B220^+^ leukemia cell chimerism in BM (Figure 3J and Supplemental Figure 3H).

To validate that our KO specifically targeted only *HoxBlinc* lncRNA and not DNA-regulatory elements embedded in its coding region, we further used shRNA to knock down *HoxBlinc* RNA in 961C cells (Supplemental Figure 3I). Knockdown (KD) of *HoxBlinc* phenocopied the antileukemic phenotypes of *HoxBlinc* KO in vitro and in vivo (Supplemental Figure 3, J–O). Thus, loss of *HoxBlinc* lncRNA decreased the leukemic burden and significantly (*P* = 0.00863, WT vs. HoxBlinc KD, Supplemental Figure 3K) attenuated NUP98-PHF23–driven leukemic progression in vivo.

### HoxBlinc loss impairs aberrant TAD integrity and the chromatin signature to rescue the NUP98-PHF23–driven leukemic transcription program.

**Figure 4:**
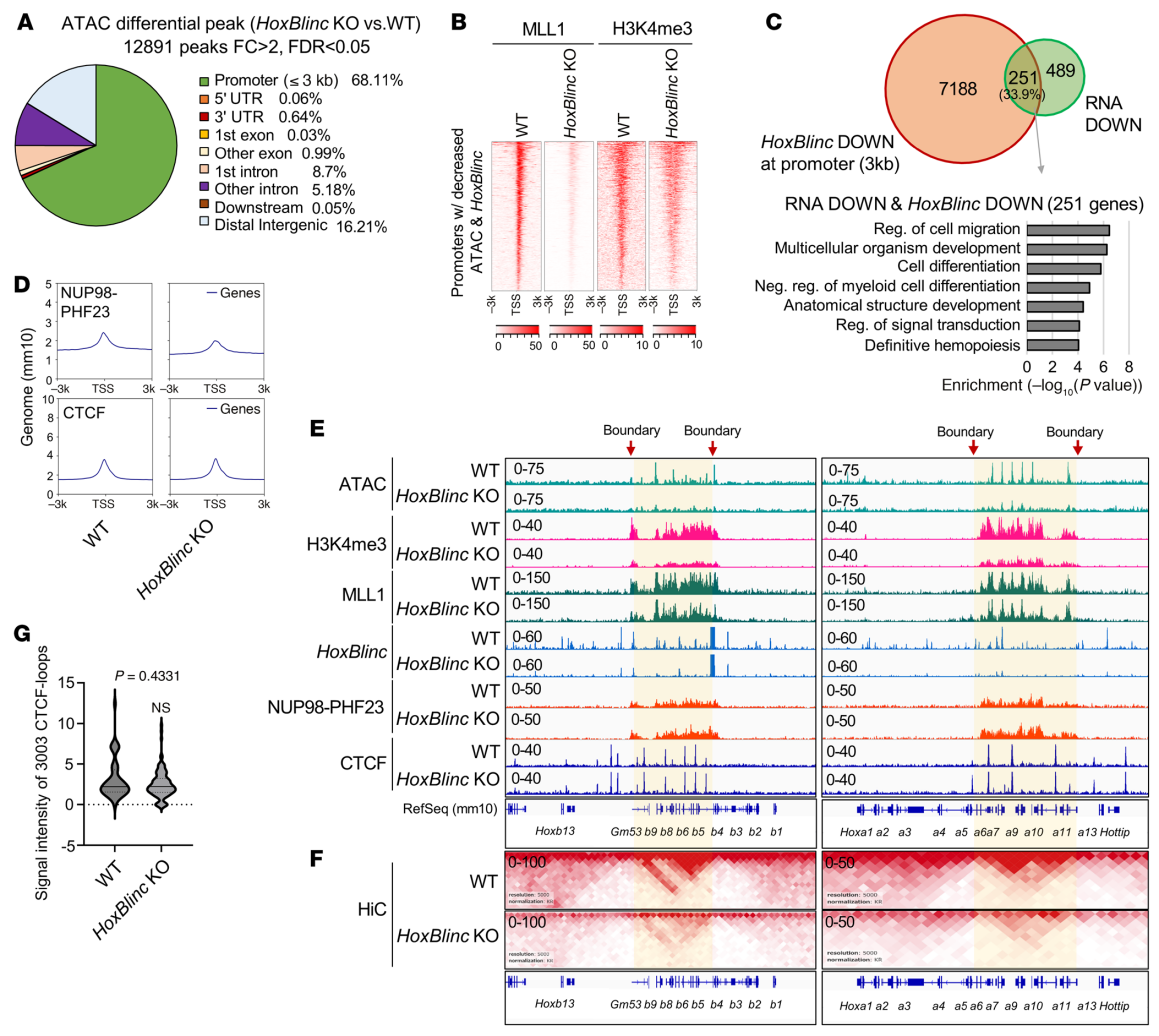
*HoxBlinc* loss impairs aberrant TAD integrity and the chromatin signature to drive the NUP98-PHF23–mediated leukemic transcription profile. (**A**) Pie chart of ATAC-Seq differential peak distribution in WT versus *HoxBlinc-*KO 961C cells. (**B**) Heatmaps showing that MLL1 recruitment/H3K4me3 at the combined *HoxBlinc-*binding and ATAC peak decreased promoter regions upon *HoxBlinc* loss. (**C**) Overlap of downregulated genes was determined by RNA-Seq and the decrease in *HoxBlinc*-binding peaks was determined by ChIRP-Seq upon *HoxBlinc* loss in 961C cells (top) and GO analysis of 251 overlapping genes (bottom). (**D**) Distribution of global NUP98-PHF23-V5 binding and CTCF binding in the mouse 961C B-ALL genome upon *HoxBlinc* loss. (**E**) ATAC-Seq, H3K4me3 CUT&RUN, *HoxBlinc* ChIRP-Seq, and MLL1, NUP98-PHF23, and CTCF ChIP-Seq analysis of changes in chromatin accessibility, H3K4me3 modification levels, MLL1 recruitment, *HoxBlinc* lncRNA, and NUP98-PHF23 and CTCF binding at *Hoxa* and *Hoxb* loci upon *HoxBlinc* depletion. *HoxBlinc*-defined (red arrows) active chromatin domains are highlighted in yellow. (**F**) HiC-Seq interacting maps in part of the mouse chromosome 6 and 11 regions of *Hoxa* and *Hoxb* loci comparing WT and *HoxBlinc-*KO 961C B-ALL cells. (**G**) Signal intensity of 3,003 CTCF-mediated chromatin loops. Data are presented as the mean ± SD; no statistical significance was found by Kolmogorov-Smirnov test.

To further investigate the underlying mechanisms by which *HoxBlinc* lncRNA regulates NUP98-PHF23–mediated oncogenic homeotic gene transcription, we carried out ChIRP-Seq, ChIP-Seq, ATAC-Seq, and Hi-C analyses in WT versus *HoxBlinc*-KO 961C B-ALL cells to examine the alterations in global *HoxBlinc* and CTCF chromatin binding, as well as the corresponding changes in TAD formation and the chromatin landscape. *HoxBlinc* loss mainly affected chromatin accessibility in promoter proximal regions (68.11%) (Figure 4A), suggesting that *HoxBlinc* acts as transcription regulator in NUP98-PHF23–driven leukemia. *HoxBlinc* is known to interact with MLL1 (22), raising the possibility that *HoxBlinc* recruits MLL1 to its targets. Integrated analysis of promoter regions with decreases in both *HoxBlinc* binding and chromatin accessibility revealed that loss of *HoxBlinc* resulted in decreases in MLL1 recruitment, H3K4me3 enrichment, and promoter chromatin accessibility in these promoter regions (Figure 4, A and B). Indeed, 33.9% of downregulated genes determined by RNA-Seq (Figure 3A) exhibited decreases in *HoxBlinc* promoter occupancies, and GO term analysis further revealed that they are involved in cell migration and differentiation, regulation of myeloid differentiation, signal transduction, and definitive hemopoiesis (Figure 4C). It was particularly intriguing that loss of *HoxBlinc* did not significantly alter global NUP98-PHF23 or CTCF chromatin binding (Figure 4D). In fact, *HoxBlinc* KO slightly enhanced NUP98-PHF23 binding in the *HoxBlinc*-defined active chromatin domain in Hoxa and Hoxb loci (Figure 4E), suggesting that *HoxBlinc* probably acts downstream of NUP98-PHF23 to activate Hox-associated homeotic oncogenic gene expression.

To further investigate whether and how *HoxBlinc* regulates the Hox chromatin signature by modulating NUP98-PHF23–associated specific 3D genome organization, we then carried out Hi-C comparing WT with *HoxBlinc*-KO 961C B-ALL cells. Consistently, *NUP98-PHF23* KI led to the formation of TADs/sub-TADs encompassing epigenetically and transcriptionally active Hoxa/b, *Meis1*, and *Kit* domains (Figure 4, E and F, and Supplemental Figure 4, A and B). *HoxBlinc* depletion resulted in near-complete disruption of TAD/sub-TAD formation at the active Hoxa/b, *Meis1*, and *Kit* domains (Figures 4F and Supplemental Figure 4B), corresponding to reduced MLL1 recruitment, decreased H3K4me3 and chromatin accessibility in these active domains (Figure 4E and Supplemental Figure 4A). Interestingly, KO of *HoxBlinc* did not significantly affect CTCF binding globally or in these loci (Figure 4, D and E). Again, the majority of CTCF-binding sites do not generally colocalize with *HoxBlinc*-binding sites in the 961C genome or in these representative loci (Figure 4E, Supplemental Figure 2D, and Supplemental Figure 4A). In particular, consistent with *HoxBlinc* depletion–mediated loss of the active TAD/sub-TAD structure, ChIRP-Seq revealed that *HoxBlinc* specifically occupied chromatin boundaries defined these active TADs/sub-TADs and that depletion of *HoxBlinc* reduced its binding at these boundaries (Figure 4E and Supplemental Figure 4A). Again, CTCF did not co-bind with *HoxBlinc* in these representative TAD/sub-TAD boundaries (Figures 4E and Supplemental Figure 4A, red arrow heads). Thus, our data indicated that *HoxBlinc* lncRNA reprogrammed NUP98-PHF23–associated specific oncogenic homeotic gene TAD/sub-TAD topology in a CTCF-independent manner. To further validate that *HoxBlinc* loss did not affect CTCF-driven chromatin loops, we performed CTCF HiChIP, an efficient genomic analysis of both CTCF binding and the CTCF-directed, genome-interacting map, comparing WT with *HoxBlinc*-KO 961C B-ALL cells. Indeed, KO of *HoxBlinc* neither affected global CTCF-directed chromatin loops (Figure 4G and Supplemental Figure 4C), nor CTCF-driven structural TADs/Sub-TADs in Hoxa/b loci (Supplemental Figure 4D). In addition, the binding of CTCF and its recruitment of cohesin in Hoxa/b chromatin regions were not altered (Figure 4E and Supplemental Figure 4, D–F), consistent with the fact that *HoxBlinc* did not interact with CTCF in *NUP98-PHF23–*rearranged 961C cells (Supplemental Figure 4G). Thus, *HoxBlinc* lncRNA regulated CTCF-independent TAD boundaries and TAD/sub-TAD formation in NUP98-rearranged leukemia.

It was reported that the N-terminal of NUP98 possesses an IDR and promotes phase-phase liquid separation to facilitate NUP98-HOXA9–driven genome organization (13, 15). We thus explored whether *HoxBlinc* lncRNA regulates NUP98-PHF23–mediated phase-phase separation in organizing transcriptional active foci in leukemic cells. We found that *HoxBlinc* loss reduced NUP98-PHF23–driven nuclear puncta formation within the nucleus (Supplemental Figure 4H). In addition, *HoxBlinc* loss led to weakened H3K4me3 and RNA polymerase II (PolII) signals that disassociated with V5-tagged NUP98-PHF23 fusion proteins (Supplemental Figure 4H). Colocalization analysis of images of V5-tagged NUP98-PHF23 fusion proteins and H3K4me3 staining indicated that *HoxBlinc* KO markedly reduced colocalization of NUP98-PHF23 and H3K4me3 within the NUP98-PHF23 fusion–driven B-ALL nucleus (Supplemental Figure 4I), supporting the idea that *HoxBlinc* lncRNA plays an important role in regulating NUP98-PHF23–directed leukemic genome organization, the chromatin landscape, and the transcription signature.

### Transgenic expression of HoxBlinc in the mouse hematopoietic compartment perturbs the HSPC subpopulation, leading to diverse leukemias.

**Figure 5:**
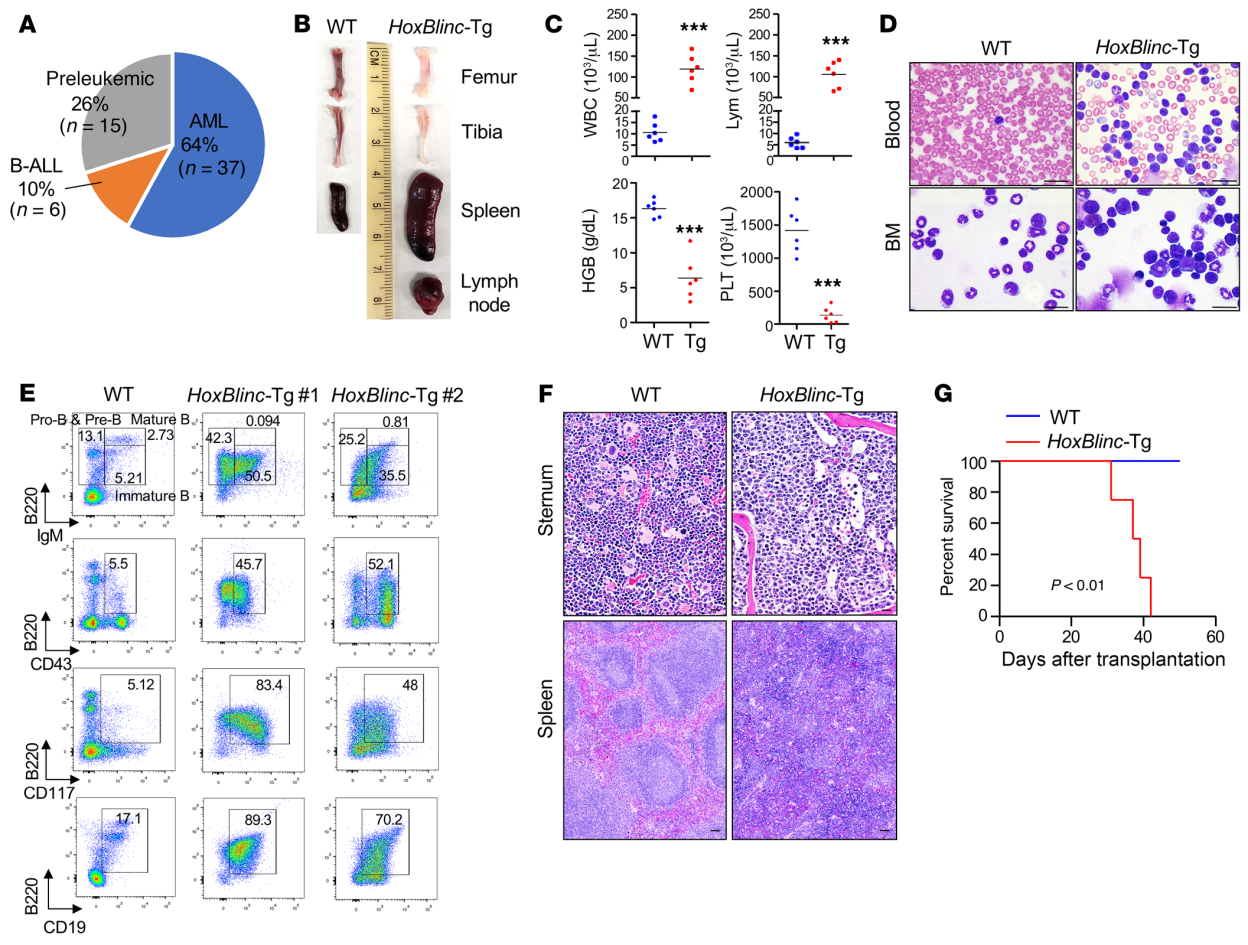
Transgenic expression of *HoxBlinc* in the mouse BM hematopoietic compartment–perturbed HSPC subpopulation leads to multiple leukemia types. (**A**) Pie chart of hematopoietic malignancies developed in 58 *HoxBlinc-*Tg mice (up to 12 months of age). (**B**) Gross appearance of BM, livers, and lymph nodes from parental mice and B-ALL *HoxBlinc-*Tg mice. (**C**) Blood count in PB from WT and B-ALL *HoxBlinc-*Tg mice. HGB, hemoglobin; PLT, platelets; Lym, lymphocytes. Data are presented as the mean ± SD. ***P ≤ 0.001, by 2-tailed, unpaired Student’s *t* test (*n* = 6). (**D**) MGG staining of PB and BM from WT and B-ALL *HoxBlinc* Tg mice. Scale bar: 50μm. (**E**) Flow cytometric analysis of B cell populations in the BM of WT and B-ALL *HoxBlinc-*Tg mice. (**F**) H&E staining of sternum and spleens from WT and B-ALL *HoxBlinc-*Tg mice. Scale bar: 50μm. (**G**) Kaplan-Meier survival curve of recipient mice receiving WT or B-ALL *HoxBlinc-*Tg splenic cells. *n* = 4/group. *P* < 0.01, by log-rank test.

The ability of *NUP98-PHF23* KI to induce various leukemias in mice depends on its ability to activate a preleukemic stem-like gene signature (17). Given that *HoxBlinc* was also able to activate the HSC/leukemic stem-like gene signature, including Hoxa, Hoxb, and *Meis1* (22), and acted downstream of the NUP98-PHF23 fusion oncoprotein (Figure 3A and Figure 4E), we then sought to determine whether overexpression of *HoxBlinc* in the mouse hematopoietic compartment partially phenocopies that in *NUP98-PHF23*–KI mice. Although the hematological phenotypes of a large cohort of aged *HoxBlinc-*Tg mice (37 of 58) exhibited abnormal hematological characteristics resembling AML (22), we found that 26% of the animals (15 of 58) did not show clear signs of hematological malignancy (preleukemic). Interestingly, a small fraction of animals (6 of 58, 10%) developed an abnormal hematopoietic phenotype closely resembling B-ALL and that was distinct from that of AML (Figure 5A). These B-ALL–like *HoxBlinc-*Tg mice had splenomegaly, enlarged lymph nodes, and pale femurs compared with WT mice (Figure 5B). PB examination revealed marked leukocytosis due to elevated lymphocyte counts, thrombocytopenia and severe anemia in these B-ALL *HoxBlinc-*Tg mice (Figure 5C). Morphologically, May-Grünwald-Giemsa–stained (MGG-stained) PB smears showed profoundly increased lymphocytic blasts (Figure 5D, top). BM cell cytospin preparations also demonstrated a predominance of lymphocyte cells (Figure 5D, bottom). Flow cytometric analyses of BM (Figure 5E) and splenic (data not shown) cells revealed predominant B220^+^ (consistently >50%) B cell populations, which were largely CD43^+^, CD117^+^, CD19^+^, and IgM^lo/–^ pre–B and immature B cells (Figure 5E). Morphologic evaluation of BM histologic sections revealed lymphoid infiltration (Figure 5F). In addition, histological evaluation of these *HoxBlinc-*Tg spleen, liver, and lymph node sections showed distortion of normal organ architecture with infiltration of lymphoid cells (Figure 5F and Supplemental Figure 5A). In addition, transplantation of 1 × 10^6^ splenic cells from a B-ALL *HoxBlinc-*Tg mouse (*HoxBlinc-*Tg 1) into sublethally irradiated recipients demonstrated that B-ALL was transferable, as all recipients developed a lethal B-ALL phenotype similar to that of the donor *HoxBlinc-*Tg mouse, e.g., marked leukocytosis evidenced by elevated lymphocytes in PB smear stains, PB lymphocyte counts, and FACS analysis (Figure 5G and Supplemental Figure 5, B–E). These data indicate that, as in *NUP98-PHF23*–KI mice, *HoxBlinc* overexpression can also cause myeloid and lymphoid malignancies.

### HoxBlinc overexpression impairs B cell development.

**Figure 6:**
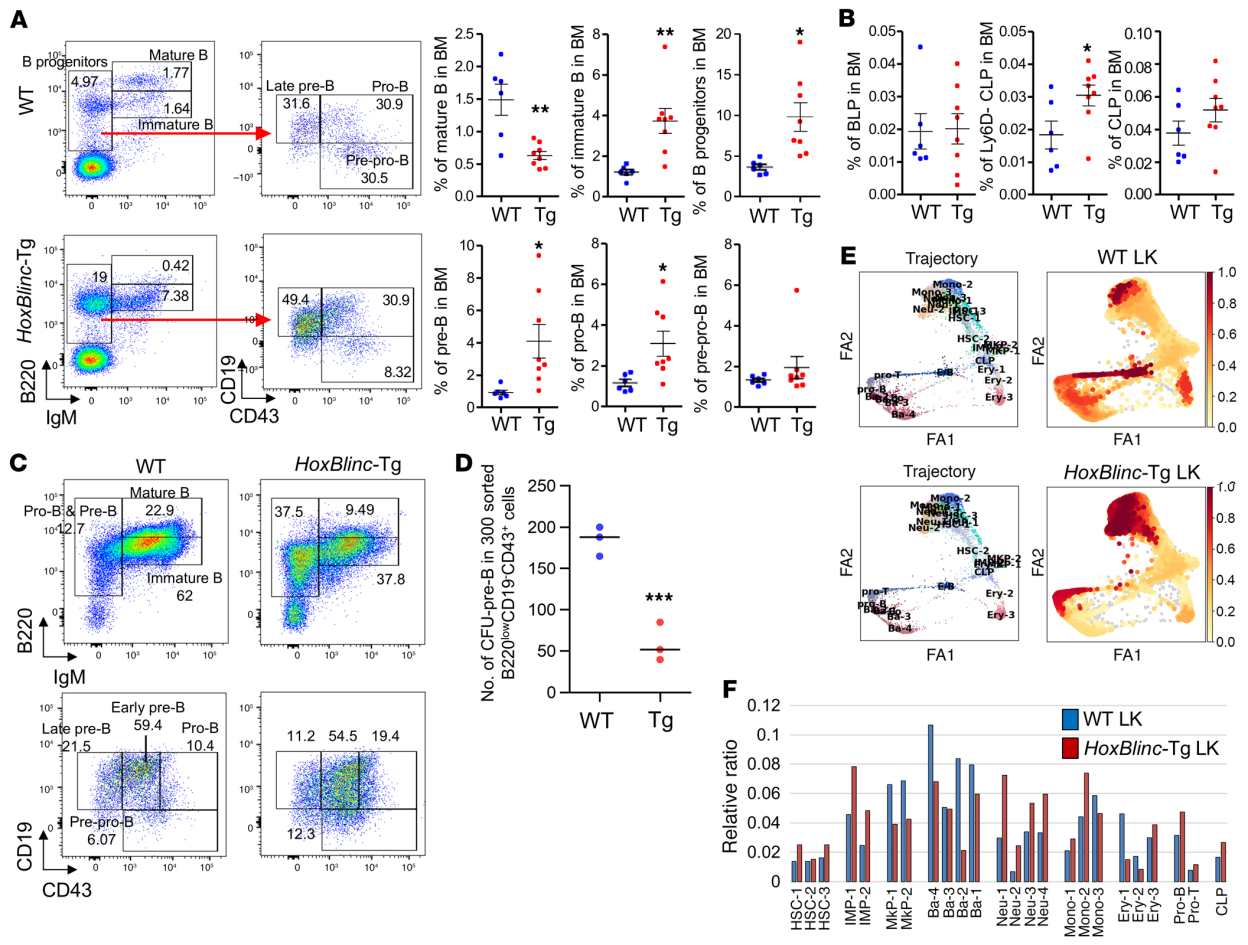
*HoxBlinc* overexpression impairs B cell development. (**A**) FACS analysis (left) and blood count (right) of B cell populations in the BM of WT (*n* = 6) and *HoxBlinc*-Tg (*n* = 8) mice. Data are presented as the mean ± SD. *P ≤ 0.05 and **P ≤ 0.01, by 2-tailed, unpaired Student’s *t* test. (**B**) Blood count of BLP and CLP populations in the BM of WT (*n* = 6) and *HoxBlinc*-Tg (*n* = 8) cells. Data are presented as the mean ± SD. *P ≤ 0.05, by 2-tailed, unpaired Student’s *t* test. (**C**) FACS analysis of B cell populations in BM cell cultures in the presence of IL-7. (**D**) Number of CFU–pre–B cells in 300 sorted B220^lo^CD19^–^CD43^+^ cells. Data are presented as the mean ± SD from 3 independent experiments. ***P ≤ 0.001, by 2-tailed, unpaired Student’s *t* test. (**E**) Trajectory inference branches/clusters were generated on the basis of expression levels of lineage-associated genes in cell clusters (left), as determined by scRNA-Seq of WT and *HoxBlinc*-Tg BM LK cells. Subpopulation cell density analysis (right) correlated with the number of enriched cells in each population. Higher cell densities are shown in dark red. (**F**) Relative ratio of HSPC subpopulations in WT and *HoxBlinc*-Tg BM LK cells, as determined by scRNA-Seq.

To further understand the cellular mechanisms by which transgenic *HoxBlinc* overexpression leads to B-ALL, we next examined the effect of *HoxBlinc* overexpression on B cell development in vivo and in vitro. Flow cytometric analyses of BM cells from young *HoxBlinc-*Tg mice (8–12 weeks old) showed dramatically increased proportions of pro–/pre–B and immature B cell populations, but decreased mature B cell proportions compared with age-matched WT mice (Figure 6A). Importantly, detailed analysis of the pro–/pre–B cell populations in WT and *HoxBlinc-*Tg BM cells revealed a significantly higher proportion of early and late pre–B, but not pro–B, cell populations in *HoxBlinc-*Tg BM cells compared with WT mice (Figure 6A). When the total number of B cell–biased lymphoid progenitor (BLP) and common lymphoid progenitor (CLP) cell populations were examined, the proportions and pools of BLPs and CLPs were comparable between WT and *HoxBlinc-*Tg mice (Figures 6B and Supplemental Figure 6A). We then examined the effect of *HoxBlinc* overexpression on B cell development by culturing WT and *HoxBlinc-*Tg BM cells in vitro in the presence of IL-7. After 1 week of culturing, *HoxBlinc-*Tg BM cells generated a significantly lower number of B220^+^ B cells than did WT BM cells, with *HoxBlinc-*Tg BM producing approximately one-third of the total cell numbers compared with the WT BM progeny cells (Figure 6, C and D, and Supplemental Figure 6B). However, the B cell progenies in the *HoxBlinc-*Tg BM cell cultures contained dramatically higher proportions of pro–/pre–B cells, but lower immature and mature B cells (Figure 6C). Further FACS analysis using anti-CD19/anti-CD43 antibody combinations revealed higher pro–B cell proportions (CD43^hi^CD19^+^) in the progenies of *HoxBlinc-*Tg BM cells (Figure 6C). Collectively, these results indicate that overexpression of *HoxBlinc* impaired B cell development by partial blockage at the pro–/pre–B cell stages.

To further explore whether and how the origin of HSPC populations that were blocked by *HoxBlinc*-Tg led to both myeloid and lymphoid malignancies, we performed single-cell RNA-Seq (scRNA-Seq) to assess the HSPC subpopulations affected by *HoxBlinc* activation *HoxBlinc*-Tg mouse LK cells exhibited biased B-lymphoid and myeloid trajectories (Figure 6E). Notably, HSC (HSC-1, HSC-3), immature myeloid progenitor (IMP-1 and IMP-2), neutrophil, monocyte, and pro–B cell subpopulations were markedly increased, while megakaryocyte, basophil, and erythroid populations were markedly reduced upon *HoxBlinc* overexpression in the hematopoietic compartment (Figure 6, E and F, and Supplemental Figure 6C). This abnormal myeloid and B cell developmental trajectory in *HoxBlinc-*Tg BM cells might have contributed to or even facilitated the pathogenesis of B-ALL and AML in the *HoxBlinc-*Tg mice.

### Overexpression of HoxBlinc activates Hox-associated TADs and the chromatin signature.

**Figure 7:**
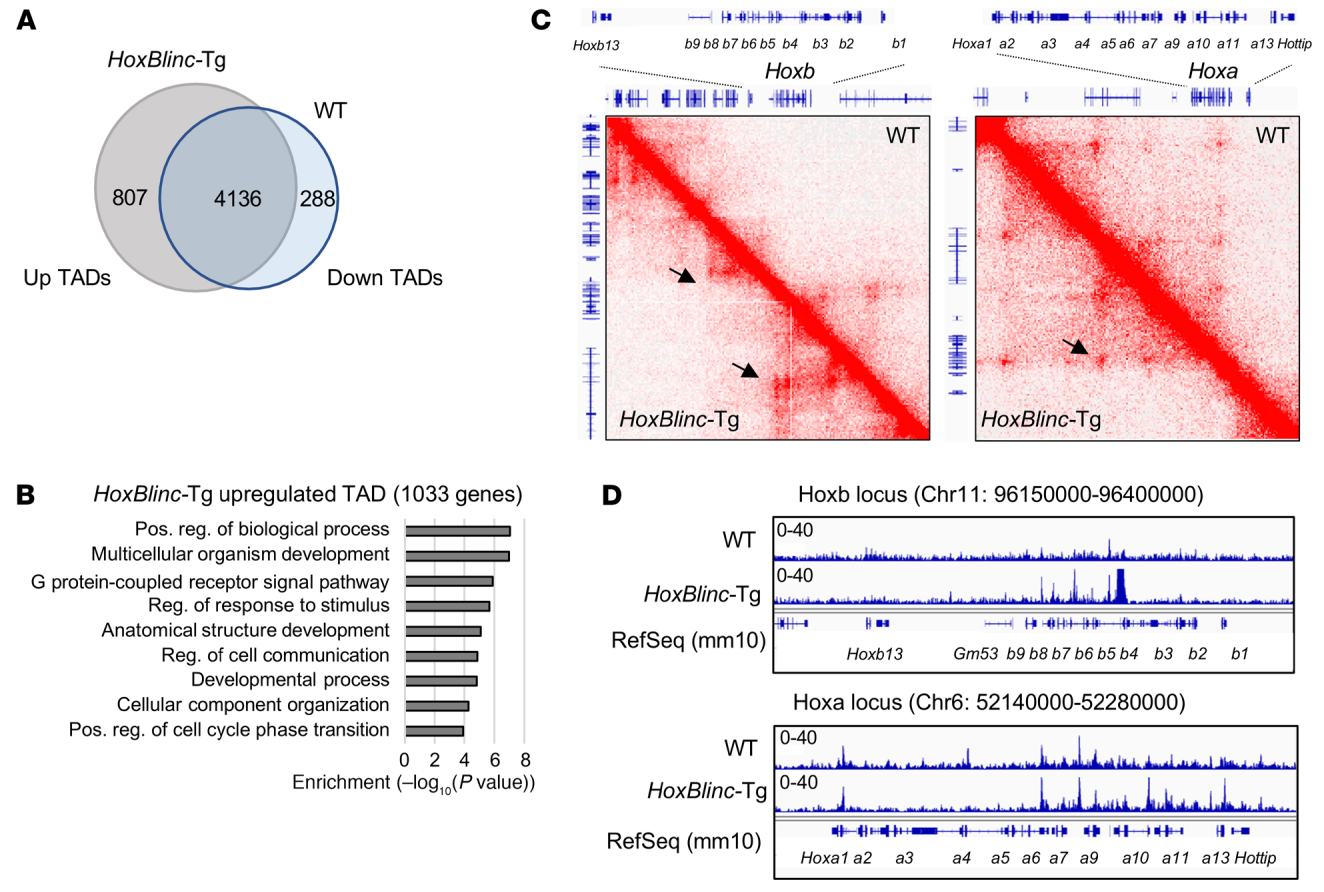
Transgenic expression of *HoxBlinc* activates leukemic stem–like gene TADs and the chromatin signature. (**A**) Pie chart shows overlapping of TADs by Hi-C in WT and *HoxBlinc*-Tg BM cells. The domain score for an altered TAD was normalized (Quantile normalization) by subtracting the mean of all TAD Hi-C signals. ANOVA was used to identify significantly altered TADs (Bonferroni-corrected *P* ≤ 0.05). (**B**) GO analysis of 1,033 genes encompassed in *HoxBlinc*-Tg upregulated TADs. (**C**) HiC-Seq interacting maps in part of mouse chromosome 6 and 11 regions of *Hoxa* and *Hoxb* loci comparing WT and *HoxBlinc*-Tg BM cells. (**D**) ATAC-Seq analysis of altered chromatin accessibility in part of mouse chromosome 6 and 11 regions of *Hoxa* and *Hoxb* loci compared WT with *HoxBlinc-*Tg BM cells.

An integrated analysis of genes downregulated upon *HoxBlinc* KO in 961C B-ALL cells and genes upregulated by transgenic expression of *HoxBlinc* in lineage Lin^–^Sca-1^+^c-Kit^+^ (LSK) cells (22) revealed that approximately 222 genes (30%) upregulated in *HoxBlinc*-Tg mice overlapped with those genes downregulated by *HoxBlin*c KO in 961C cells (Supplemental Figure 7A). These overlapping genes are mainly involved in cell differentiation, embryonic skeletal system development, multicellular organismal development, and anterior/posterior pattern specification (Supplemental Figure 7B). Thus, it is conceivable that *HoxBlinc*-Tg may be sufficient to aberrantly reorganize homeotic oncogene TADs and remodel the *Hox* gene–associated chromatin landscape to perturb normal hematopoietic transcription and HSPC function. To test this possibility, we performed Hi-C and ATAC-Seq in BM cells isolated from WT versus *HoxBlinc*-Tg mice. Transgenic expression of *HoxBlinc* in BM cells indeed altered a subset of genome TAD topology, with 807 enhanced TADs and 288 reduced TADs (Figure 7A). GO analysis of genes encompassed in upregulated TADs revealed that these genes (*n* = 1,033 genes) were involved in pathways regulating biological/developmental processes, cell communication, and cell-cycle phase transition (Figure 7B). In particular, overexpression of *HoxBlinc* markedly enhanced TADs in *Hoxa*, *Hoxb*, and *Kit* loci (Figures 7C and Supplemental Figure 7C). ATAC-Seq analysis consistently showed that chromatin accessibility was dramatically increased in *Hoxa*, *Hoxb*, and *Kit* loci as well (Figures 7D and Supplemental Figure 7D). Together, our data indicated that transgenic expression of *HoxBlinc* aberrantly enhanced oncogenic homeotic TAD structure and chromatin accessibility, resulting in an oncogenic homeotic gene expression signature and development of leukemia.

### HOXBLINC is required for NUP98-HOXA9–driven homeotic gene transcription and leukemic transformation.

**Figure 8:**
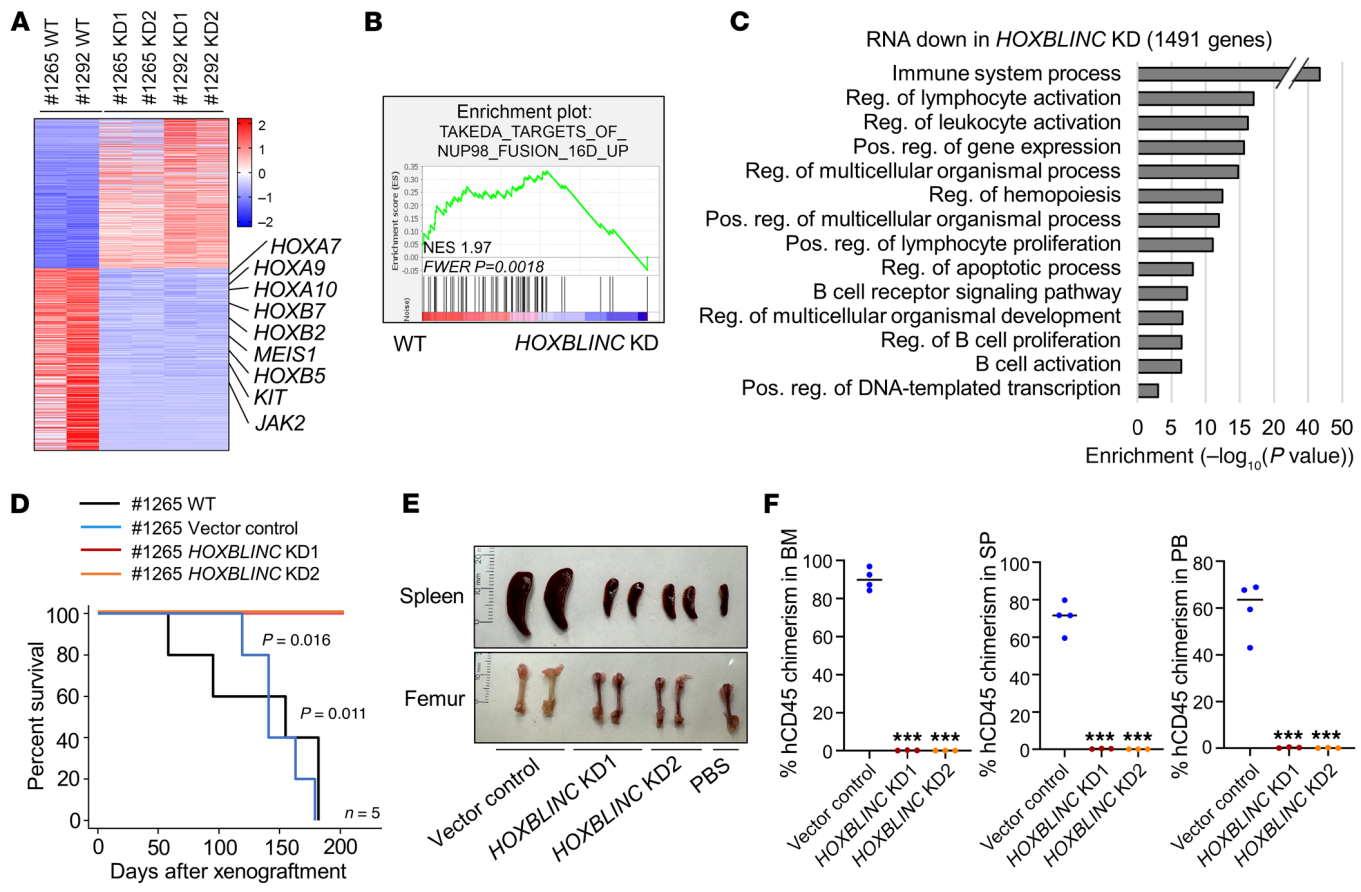
*HOXBLINC* lncRNA is also required for NUP98-HOXA9–driven homeotic gene transcription and leukemic transformation. (**A**) Heatmap of DEGs upon *HOXBLINC* KD in primary AML cells from 2 patients carrying the NUP98-HOXA9 fusion. (**B**) GSEA of DEGs upon *HOXBLINC* KD in NUP98-HOXA9 fusion–driven AML patient samples (patients 1265 and 1292) using the TAKEDA_TARGETS_OF_NUP98_HOXA9_FUSION_16D_UP (M15588) gene set. (**C**) GO analysis of downregulated genes in NUP98-HOXA9 fusion–driven AML patient samples (patients 1265 and 1292) upon *HOXBLINC* KD. (**D**) Kaplan-Meier survival curve for PDX mice that received WT, vector control, or *HOXBLINC-*KD AML cells from patient 1265. *P* = 0.016 for the vector control versus KD; *P* = 0.011 WT versus KD, by log-rank test followed by Bonferroni’s post hoc test (*n* = 5). (**E**) Images of spleens and femurs from NSGS mice 143 days after transplantation of WT, vector control, or *HOXBLINC-*KD primary AML cells from patient 1265 carrying the NUP98-HOXA9 fusion. (**F**) hCD45^+^ cell chimerism in BM/PB/SP of NSGS mice 143 days after transplantation of vector control (*n* = 4) and 2 independent *HOXBLINC-*KD cells from patient 1265 with primary AML (*n* = 3/clone) carrying the NUP98-HOXA9 fusion. Data are presented as the mean ± SD. *P ≤ 0.05, **P ≤ 0.01, and ***P ≤ 0.001, by ANOVA following a Bonferroni post hoc test.

NUP98 fusion oncoprotein–driven leukemogenesis is mediated by alteration of chromatin domain structures and expression of a HOX/homeotic gene–associated oncogenic signature (6). To further test whether *HOXBLINC* is generally required for mediating the NUP98 fusion oncoprotein–driven HOX/homeotic oncogene transcription and leukemogenesis, we measured *HOXBLINC* lncRNA expression levels in patients with AML carrying a NUP98-HOXA9 translocation (patients 1265 and 1292) and compared with levels in the *HOXBLINC*-expressing OCI-AML3 cell line (Supplemental Figure 8A). Both patient 1265 and patient 1292 showed significantly higher *HOXBLINC* expression levels than did OCI-AML3 cells, while OCI-AML2– and MLL-AF9–translocated patient samples did not express *HOXBLINC* (Supplemental Figure 8A). To further examine the role of *HOXBLINC* in NUP98-HOXA9–driven aberrant gene expression patterns and leukemogenesis, we inhibited *HOXBLINC* RNA expression using a lentivirus with shRNA-mediated gene silencing (Supplemental Figure 8B) and conducted RNA-Seq analysis of WT and *HOXBLINC*-KD in primary AML cells from patients 1265 and 1292 harboring the NUP98-HOXA9 fusion. KD of *HOXBLINC* in primary NUP98-HOXA9 AML cells resulted in significantly downregulated HOX/homeotic oncogenic gene signatures, including for *HOXA9*, *A10*, *B5*, *MEIS1*, *KIT*, and *JAK2* (Figure 8A). GSEA revealed that the top ranked pathway impaired by *HOXBLINC* KD was the NUP98-HOXA9 fusion gene signature (Figure 8B). GO analysis consistently indicated that the pathways affected by *HOXBLINC* KD were those involved in immune system processes, leukocyte/lymphocyte activation and proliferation, hemopoiesis, and transcriptional regulation (Figure 8C).

Finally, we transplanted 1 × 10^6^ WT, vector control, or *HOXBLINC*-KD primary AML cells from patient 1265 into NSG mice. The mice that received WT or vector control NUP98-HOXA9 AML cells became moribund and/or died 58–182 days after transplantation, whereas the mice that received *HOXBLINC*-KD cells remained healthy for at least 200 days (Figure 8D). Similarly, *HOXBLINC* KD in patient 1292 AML cells also significantly prolonged the survival of the transplanted mice (Supplemental Figure 8C), indicating that loss of *HOXBLINC* inhibited the leukemogenic potential of NUP98-HOXA9 leukemic cells. The moribund mice that received the NUP98-HOXA9 AML cells developed leukemia, as evidenced by splenomegaly, pale tibias, and markedly increased numbers of immature myeloid cells in PB and BM as compared with mice that received *HOXBLINC*-KD cells or the PBS control (Figure 8E and Supplemental Figure 8D). Finally, FACS analysis revealed that loss of *HOXBLINC* eliminated human CD45^+^ leukemic cells from BM, PB, and spleen (Figure 8F). Thus, *HOXBLINC* lncRNA was essential for maintaining the HOX/homeotic oncogenic transcription profile and leukemogenesis driven by NUP98 fusion oncoproteins, perhaps by reprograming CTCF-independent TAD topology and the chromatin signature.

## Discussion

### The role of HoxBlinc lncRNA in NUP98 fusion–associated genome reorganization and oncogenic transcription.

In humanized *NUP98-PHF23* and *NUP98-HOXA9*–KI mice, it has been reported that NUP98 fusion drives leukemogenesis by altering chromatin structure and gene expression (15, 17, 18), given that homeodomain-containing fusion partners are known to have a role in epigenetic and transcriptional regulation. It was speculated that the 3D genome organization and gene transcription driven by NUP98 fusions depend on the fusion partner’s ability to recognize the H3K4me3 region or induce phase-phase separation (13, 15, 17, 18). In addition to its fusion partners, NUP98 itself has an ability to recruit the Wdr82–Set1A COMPASS (WSC) complex to regulate H3K4me3 patterns and promote hematopoietic gene expression (10). Thus, the molecular basis underlying NUP98 fusion–mediated homeotic oncogene activation, HSPC perturbation, and, eventually, leukemogenesis remains largely elusive. In the current studies, we provide several lines of evidence demonstrating that *HoxBlinc* lncRNA acted as an oncogenic regulator downstream of NUP98-PHF23 to organize TAD topology and expression of HSC/leukemia-associated genes including *Kit*, posterior *Hoxa*, middle *Hoxb*, and *Meis1*. First, *HoxBlinc* bound to TAD boundaries and promoters of HOX and other homeotic oncogene loci in NUP98-PHF23 leukemic cells. Second, loss of *HoxBlinc* lncRNA did not affect NUP98-PHF23 chromatin binding. Third, *HoxBlinc* loss in NUP98-PHF23 leukemic cells impaired HOX/homeotic signature gene TADs, chromatin structure, and gene expression, thereby mitigating NUP98 fusion–driven leukemogenesis. Last, transgenic expression of *HoxBlinc* lncRNA in mouse hematopoietic compartment promoted leukemic TAD topology, chromatin accessibility, and homeotic oncogene transcription, leading to various leukemias resembling NUP98-PHF23–driven leukemic phenotypes. Thus, our studies reveal a key role of *HoxBlinc* lncRNA in NUP98 fusion–driven leukemic TAD organization and oncogenic gene-regulatory networks.

### The roles and treatment potential of HoxBlinc lncRNA in hematopoietic malignancies.

LncRNAs often exhibit tissue- and lineage-specific expression patterns and play essential roles in regulating lineage-specific differentiation including hemopoiesis (20, 23). *HoxBlinc* lncRNA was originally identified as an activator of anterior/middle *Hoxb* genes during embryonic hematopoietic development (21). To facilitate anterior/middle *Hoxb* gene expression, *HoxBlinc* lncRNA associates with SETD1A-MLL1 complexes and the H3K4me3 at the promoters (21, 22). LncRNAs exhibit great versatility in their mechanism of action, influencing many nuclear processes, such as spatial conformation of chromosomes, histone modifications, and gene transcription (23). Although many cancer-associated lncRNAs have been shown to play an important role in specific oncogene-associated gene expression (24–26), it remains unclear in the majority of cases whether lncRNA itself is sufficient to drive oncogenesis or leukemogenesis. The fact that transgenic expression of *HoxBlinc* enhanced HSPC self-renewal and led to the development of diverse leukemias in mice, reminiscent of NUP98-PHF23–driven leukemogenesis, indicates an oncogenic role of *HoxBlinc* lncRNA in NUP98 fusion–driven leukemogenesis. In *NPM1^C+^* AML, *HOXBLINC* lncRNA also acts as a downstream mediator to achieve the NPM1^C+^-associated HOX transcription signature and leukemogenesis (22). Similar to NPM1^C+^, NUP98 fusion also aberrantly activated both *HOXA* and *HOXB* genes to facilitate leukemogenesis. It is conceivable that *HOXBLINC* plays an important role in HOXA/B homeobox oncogene transcription and leukemogenesis. However, it is worth noting that posterior HOXA locus–associated lncRNA *HOTTIP*, which is highly expressed in AML carrying *MLL* rearrangements or the *NPM1^C+^* mutation (27), regulates predominantly posterior *HOXA* genes, but not the *HOXB* locus. *HOTTIP* coordinates posterior *HOXA* TAD organization to drive oncogenic *HOXA* gene expression, e.g., *HOXA9* and *HOXA10*, and promotes HSC/LSC aberration in myeloid malignancy. It remains to be investigated whether *HOTTIP* also plays a role in NUP98 fusion oncoprotein–driven *HOXA* gene transcription and whether *HOXBLINC* and *HOTTIP* coordinate to promote the expression of *HOX* and other homeotic oncogenes, resulting in diverse leukemias.

Given that lncRNAs, such as *HOXBLINC* and *HOTTIP*, play an oncogenic role in specific subtypes of AML and B-ALL, these oncogenic lncRNAs become attractive targets for the treatment and diagnosis of cancers including leukemia. Various RNA-based approaches, such as antisense oligonucleotides (ASOs), siRNAs, or miRNA mimics and sponges, have been proposed to target disease- and oncogene-driven noncoding RNAs (28, 29). However, their efficient delivery into particular cell populations to achieve specific targeting remains a daunting challenge, especially for therapeutic purposes. CpG oligonucleotide–conjugated siRNAs targeting *Stat3* or *miR-126* specifically exert an antileukemic effect, suggesting their potential clinical application (30, 31). Refined CRISPR/Cas9/Cas13-mediated genome or direct transcript editing of lncRNAs, as well as targeting of the RNA-mediated structure or complex (e.g., the R-loop complex) at specific genomic locations can also be used to modulate lncRNA expression and function (32). The advances in new RNA-centric technologies will not only lead to mechanistic insights into lncRNA-driven oncogenesis, but also the development of potential therapeutic options for lncRNA-associated diseases. Given that lncRNAs, such as *HOTTIP* and *HOXBLINC*, are expressed in a cell-type/AML subtype–specific fashion (22, 27), targeting this class of lncRNAs may specifically eliminate homeotic oncogenic pathways and lead to the eradication of specific leukemias.

### The mechanism of HoxBlinc lncRNA–mediated oncogenic TAD formation.

Different subtypes of AML form specific TADs and sub-TADs that underlie subtype-specific rewiring of gene-regulatory networks and oncogenic gene expression (33, 34). Although TADs are functionally important in gene expression, it is difficult to assess how they are dynamically organized to facilitate the subtype- and lineage-specific gene-regulatory circuit and transcription and whether the conformation effects are a cause or consequence of gene regulation Many studies have revealed that CTCF and its associated cohesin complex constitute a master regulator of mammalian genome organization (35–39). CTCF’s genome functions require a cohesin complex, which catalyzes the folding of the genome into loops that are anchored by CTCF (40, 41). Indeed, cohesin is essential for CTCF-anchored TAD boundary formation by trapping the progressive and dynamic movement DNA loop into its ring-like structure and becoming stalled in the TAD boundary bound by CTCF, a so-called loop extrusion, thereby influencing chromatin structure dynamics including the formation or stabilization of chromatin loops (42, 43). In the case of NUP98-rearranged leukemia cells, NUP98-HOXA9 fusion–mediated phase separation induced CTCF-independent chromatin loops (15). Intrinsic and regulated phase separation has been linked to chromatin/genome organization and gene transcription (44–47). Given that most NUP98 fusions harbored IDR and that *HoxBlinc* loss impaired the colocalization of NUP98-PHF23 with H3K4me3 in the nucleus and NUP98 fusion–associated TAD topology (Figure 4 and Supplemental Figure 4H) without affecting global NUP98-PHF23 and CTCF binding, there are 2 likely scenarios. First, *HoxBlinc* lncRNA is involved in the regulation of NUP98 fusion–mediated phase separation, leading to CTCF-independent TAD rewiring of Hox/homeotic oncogene loci. Second, unlike *HOTTIP* lncRNA–dependent CTCF/cohesin boundaries in *MLLr^+^* driven AML (32), *HoxBlinc* may associate with other chromatin boundary proteins other than CTCF to reinforce TAD formation in leukemic cells carrying NUP98 fusions. Thus, it is warranted to determine the *HoxBlinc* RNA–associated proteomic complexes, which will enable us to further understand the mode of action of *HoxBlinc* lncRNA in NUP98 fusion–driven leukemogenesis and to identify novel chromatin-TAD boundary complexes.

## Methods

### Sex as a biological variable.

Our study used both male and female mice.

### Samples from patients with AML.

Samples from patients with primary patient AML who harbored a NUP98-HOXA9 translocation (nos. 1265 and 1292) were collected.

### HoxBlinc-transgenic mice.

*HoxBlinc*-transgenic mice have been previously described (22). Full-length mouse *HoxBlinc* cDNA was subcloned into the HS321/45-vav vector31 by SfiI and NotI so that the transgene was located between the Vav1 promoter and Vav1 enhancer.

### Cell lines.

BaF3 cells were cultured in IMDM supplemented with heat-inactivated FBS, 10 ng/mL mouse recombinant IL-3 (78042, STEMCELL Technologies), and penicillin/streptomycin. Mouse primary B-ALL cells harboring NUP98-PHF23 fusion, 961C, and A1929 cells were generated from *NUP98-PHF23*–KI mice and described previously (17). 961C cells were maintained in IMDM supplemented with heat-inactivated FBS and penicillin/streptomycin (17). A1929 cells were maintained in RPMI supplemented with 10% FBS, 50 μM 2-mercaptoethanol (21985023, Gibco, Thermo Fisher Scientific), and 10 ng/mL mouse recombinant IL-7 (78054, STEMCELL Technologies) (48).

For *HoxBlinc* KO, we transfected 961C or A1929 cells with 1 μg epiCRISPR (49) (Addgene no. 135960) harboring sgRNA-targeted mouse *HoxBlinc* by Lipofectamine 3000 (L3000-008, Thermo Fisher Scientific) according to the manufacturer’s instructions. After changing to fresh media, the cells were incubated in a CO_2_ incubator for 2 days. Transfected cells were treated with 1 μg/mL puromycin for selection.

### RNA extraction, reverse transcription quantitative PCR, RNA-Seq, and data analysis.

RNA was extracted using the Quick-RNA Miniprep kit (R1054, Zymo Research) according to the manufacturer’s protocol. cDNA was generated from 1 μg RNA by High-Capacity RNA-to-cDNA Kit (4387406, Thermo Fisher Scientific) and used as a template for reverse transcription quantitative PCR (RT-qPCR).

RNA (1 μg) was used for RNA-Seq library preparation with the NEBNext Ultra II Directional RNA Library Prep kit (E7765, New England Biolabs) according to the manufacturer’s protocol. RNA-Seq analysis is described in the Supplemental Methods.

### ATAC-Seq library preparation.

An ATAC-Seq assay was performed using the Nextera DNA library preparation kit (20034210, Illumina) according to the manufacturer’s protocol and as previously described (27). ATAC-Seq analysis is described in Supplemental Methods.

### ChIP assay and library preparation.

ChIP assays were performed as described previously (50). Immunoprecipitated DNA (10 ng) was used as input for the library preparation using the NEBNext Ultra II DNA Library Prep Kit for Illumina (E7645S, New England Biolabs). Libraries were subjected to paired-end sequencing at a 150 bp length on an Illumina NovaSeq 6000. Details on the ChIP-Seq processing and analysis are described in Supplemental Methods.

### Chromatin isolation by RNA purification assays and library preparation.

ChIRP assays were performed as described previously (22). A ChIRP-Seq library was prepared using the NEBNext Ultra II DNA Library Prep Kit for Illumina (E7645S, New England Biolabs). Libraries were subjected to paired-end sequencing at a 150 bp length on an Illumina NovaSeq 6000. Details regarding ChIRP-Seq processing and analysis are described in the Supplemental Methods.

### Cleavage under targets and release using nuclease assay.

Cleavage under targets and release using nuclease (CUT&RUN) assays were performed using with CUTANA ChIC/CUT&RUN Kit (14-1048, EpiCypher) according to the manufacture protocol. Details are described in the Supplemental Methods.

### High-throughput chromosome conformation capture assay.

A high-throughput conformation capture (Hi-C) assay was performed using Arima-HiC+ Kit (A410030, Arima Genomics) according to the manufacturer’s protocol as previously described (27, 32). Details regarding HiC-Seq processing and analysis are described in the Supplemental Methods.

### Transplantation.

Adult B6.SJL-*Ptprc*^a^ *Pepc*^b^/BoyJ mice (002014, The Jackson Laboratory, 8–12 weeks old) were treated with 4 doses of busulfan and transplanted in each test group with 1 × 10^6^ 961C or A1929 cells by tail-vein injection. Fourteen days for 961C or 23 days for A1929 after transplantation, PB was collected and depleted of RBCs by ammonium chloride (420301, BioLegend). BM was isolated from tibias and femurs and filtered using a 70 μm filter (Corning). Mouse CD45.2 chimerism in the BM and PB cells was analyzed with anti–mouse CD45.1 (17-0453-82, clone A20, BioLegend) and anti–mouse CD45.2 (12-0454-82, clone 104, BioLegend) antibodies with a BD Accuri C6 Plus flow cytometer.

### MGG staining.

Air-dried PB smear slides from mice were incubated with May-Grünwald Giemsa Staining solution (MG500, MilliporeSigma) for 3 minutes at room temperature and rinsed with PBS, pH 6.7. the slides were stained with Giemsa working solution (20× dilution with PBS pH 6.7, GS500, MilliporeSigma) for 15 minutes at room temperature. After washing with distilled water, the slides were air dried and mounted with VectaMount Permanent Mounting medium (Vector Laboratories).

### Immunofluorescence.

Cells (0.5 M) were resuspended in 400 μL 1% BSA in PBS and centrifuged at 700 rpm for 4 minutes using Cytospin 4 (Thermo Fisher Scientific). The slides were fixed with 4% PFA in PBS (Santa Cruz Biotechnology) for 15 minutes and permeabilized with 0.1% Tween-20 for 15 minutes. After blocking in 1% BSA in PBS, the slides were incubated with the primary antibodies anti-V5 (R960-25, clone SV5-Pk1, Invitrogen, Thermo Fisher Scientific), anti–Pol II (14-9802-82, clone 3E10, Invitrogen, Thermo Fisher Scientific), and anti-H3K4me3 (9751, clone C42D8, Cell Signaling Technology) at 4°C overnight. The slides were washed in PBS and incubated with Alexa Fluora 488 donkey anti–mouse IgG, Alexa Fluora 555 donkey anti–rat IgG, and Alexa Fluora 594 donkey anti–rabbit IgG (A-21202 and A78945, A21207, Invitrogen, Thermo Fisher Scientific). After washing 3 times in PBS, the slides were mounted using ProLong Diamond Antifade Mountant with DAPI (P36962, Invitrogen, Thermo Fisher Scientific). A Leica SP8 STED 3× Inverted Confocal microscope was used for imaging.

### HSPC sorting.

HSPCs from WT and *HoxBlinc*-Tg mice were sorted as previously described (22). Briefly, Lin^+^ BM cells were depleted from the BM of 8- to 12-week-old mice using Miltenyi Biotec magnetic beads (130-110-470, Miltenyi Biotech.). Subsequently, Lin^–^ BM cells were stained with lineage, Sca-1, and c-kit antibodies. Lin^–^Kit^+^ (LK) cells were sorted using a BD FACSAria II flow cytometer. The purity of selected LSK cells was routinely over 98%. Data were analyzed with FlowJo software (version 10). The antibodies used for FACS are listed in Supplemental Table 1.

### scRNA-Seq.

Single cells were generated using Chromium Controller (10x Genomics), and scRNA-Seq libraries were constructed using chromium single-cell 3′ reagent kits v2 (10x Genomics) according to the manufacturer’s recommendations. Data analysis was performed as previously described (32), and details are described in Supplemental Methods.

### Xenotransplantation of human leukemic cells and patient-derived xenografts.

Adult NOD.Cg-*Prkdc*^SCID^*Il2rg*^tm1Wjl^ Tg(CMV-IL3,CSF2,KITLG)1Eav/MloySzJ (013062, The Jackson Laboratory, 8–12 weeks old) mice were pretreated with 2 doses of busulfan (20 mg/kg/day). Busulfan-treated mice were transplanted with 1 M cells of primary AML patient cells by tail-vein injection.

PB was collected 143 days after transplantation and depleted of RBCs by ammonium chloride treatment, BM was isolated from the femurs, and the spleen was processed into a single-cell suspension. Human CD45^+^ chimerism in the BM, spleen, and PB cells was analyzed with the BD Accuri C6 Plus flow cytometer.

### Statistics.

For in vitro experiments, at least 3 independent experiment with at least 3 biological replicates for each condition/genotype were carried out. GraphPad Prism (version 10.3.1, GraphPad Software) was used for statistical analysis and graph generation. Statistical differences were determined by 2-tailed, unpaired Student’s *t* test or 1-way ANOVA followed by Bonferroni’s post hoc analysis for 2-group or multigroup comparisons, respectively. *P* values for CTCF-associated loops from CTCF HiChIP were calculated by Kolmogorov-Smirnov test. Kaplan-Meier analysis with a log-rank test were performed for overall survival comparison in transplantation or patient-derived xenograft (PDX) experiments. The sample size of 5 mice/group/genotype containing both male and female animals at similar ages (8–12 weeks of age was chosen, and animals were randomly assigned to each study arm). A *P* value of less than 0.05 was considered statistically significant.

### Study approval.

Transplantation and xenograft studies were approved by and conducted in accordance with the regulatory guidelines of the Penn State Hershey Medical Center. *HoxBlinc*-transgenic mice studies were approved by and conducted in accordance with the regulatory guidelines by the IACUC of UT Health San Antonio. The acquisition of samples from patients with primary AML who harbored a NUP98-HOXA9 translocation (nos. 1265 and 1292) were approved by the IRB of the Pennsylvania State University College of Medicine in accordance with the Declaration of Helsinki.

### Data availability.

All data that used for analyses in this study can be requested from the corresponding author. All next-generation sequencing data were uploaded to the Gene Expression Omnibus (GEO) database (GEO GSE269258). Values for all data in graphs are reported in the Supporting Data Values file.

## Author contributions

KH, YQ, PDA, and SH conceived of and designed the experiments. KH, GZ, QL, JL, and NN performed experiments. HL performed bioinformatics and statistical analysis. KH, JL, GZ, QL, YL, FCY, NN, MX, and PDA established the transplantation/KI/transgenic mouse models. NN, MX, AS, DC, and PDA provided critical reagents and patient sample analyses. SH, YQ, and MX wrote and edited the manuscript. SH supervised the studies.

## Supplementary Material

Supplemental data

Unedited blot and gel images

Supporting data values

## Version 1 01/30/2025

In-Press Preview

## Version 2 04/01/2025

Electronic publication
