# Supplementary material for: HoxBlinc lncRNA reprograms CTCF-independent TADs to drive leukemic transcription and HSC dysregulation in NUP98-rearranged leukemia
Source: J Clin Invest. 2025 Jan 30;135(7):e184743. doi: 10.1172/JCI184743 (PMC11957699; doi:10.1172/JCI184743)
Supplement: Supplemental data [file jci-135-184743-s233.pdf]

Supplemental Material

Supplemental Methods

RNA-seq analysis

Quality of RNA-seq library was assessed by Qubit and Bioanalyzer. Libraries were subjected to paired-end sequencing at a 150 bp length on an Illumina NovaSeq 6000. Sequence reads were trimmed and quality filtered using cutadapt (version 1.18) (1) and aligned to human (hg19) or mouse genome (mm10) using HISAT2 (version 2.2.1) (2). The read counts were calculated by featureCounts (version 2.0.3) (3). Normalized counts, fold changes and p-value between cell condition were generated by DEseq2 (version 1.40.2) (4). Read count files were deposited in the NCBI GEO under accession number (GEO: GSE269258).

ATAC-seq analysis

Libraries were subjected to 150 bp paired-end sequencing on an Illumina NovaSeq 6000. Sequence reads were trimmed and quality filtered using cutadapt (version 1.18) (1) and aligned to human (hg19) or mouse genome (mm10) using Bowtie2 (version 2.5.2) (5) with parameters "--very-sensitive -X 1000 –dovetail". Reads with low mapping quality (mapQ<30), reads that are not properly paired and mitochondrial reads were removed using bamtools (version 2.5.1) (6). PCR duplicates were removed using Picard MarkDuplicates (http://broadinstitute.github.io/picard, version 2.26.10). Filtered alignment BAM files were converted to BED files. Peak calling was performed using MACS2 (version 2.2.6) (7) with parameters "--qvalue '0.05' --keep-dup 'all' --d-min 20 --buffer-size 100000 --bdg -f BED --nomodel --extsize '200' --shift '-100' ". Treatment bedgraph is converted to bigWig file using bedGraphToBigWig for visualization. ATAC-seq tracks were viewed using the Integrated Genomic Viewer (version 2.8.13) (8). Differential ATAC peaks were identified using DiffBind (version 2.10.0) (9) and followed ChIPseeker (version 1.28.3) (10) for annotation. Bigwig files

and annotated peak files were deposited in the NCBI GEO under accession number (GEO: GSE269258).

Chromatin immunoprecipitation (ChIP) assay

Briefly, $1x10^7$ cells were collected and cross-linked by 1% formaldehyde (BP531, Fisher Bioregents) and quenched by 125mM glycine. Crosslinked cell pellet was resuspended by ChIP lysis buffer and subjected to chromatin shearing using Bioruptor (Diagenode) with 30s on and 30s off condition. Sonicated chromatin was incubated with anti-CTCF (2899, Clone: N/A, Cell Signaling Technology), anti-V5 (R960-25, Clone: SV5-Pk1, Invitrogen) or anti-MLL1 (A300-086A, Clone: N/A, Bethyl Laboratories) antibody at 4°C overnight. Antibody/Protein beads bounded chromatin was washed 5 times and reverse-crosslinked. Immunoprecipitated DNA was purified and analyzed by qPCR to quantify relative enrichment of the target loci. Libraries were generated using NEBNext Ultra II DNA Library Prep Kit for Illumina (E7645S, New England Biolabs) and subjected to 150 bp paired-end sequencing on an Illumina NovaSeq 6000.

Chromatin isolation by RNA purification (ChIRP) assay

Briefly, $1x10^8$ cells were cross-linked in PBS with 1% formaldehyde (BP531, Fisher Bioregents) for 10min at room temperature with rotating and quenched by 125mM glycine for 5min. After washing cell pellet by ice-chilled PBS, the pellet was lysed in 1ml of lysis buffer (50 mM Tris-HCl pH 7.0, 10 mM EDTA, 1% SDS, add PMSF, PIC and Superase-in in fresh) per 100mg of cell pellet. Chromatin was fragmented by sonication with Bioruptor UCD200 (Diagenode) and cleaned up by centrifuge (16100G, 15min, 4°C). Cleared chromatin was diluted in 2X volume of freshly-made hybridization buffer (750 mM NaCl, 1% SDS, 50 mM Tris-HCl pH 7.0, 1 mM EDTA, 15% formamide, DTT, PMSF, P.I, and Superase-in) and hybridized with 100pmol of biotinylated DNA probes targeting *HoxBlinc* or *LacZ* at 37°C overnight. DNA/RNA hybrids were precipitated using 100μL of Streptavidin-magnetic C1 beads (65001,

Invitrogen) and washed 5 times with washing buffer (2x SSC, 0.5% SDS). RNA was isolated using TRIzol reagent (93289, Sigma-Aldrich) and purified with ZYMO RNA concentrator kit (R1013, ZYMO research). *HoxBlinc* RNA retrieval was confirmed by RT-qPCR with GAPDH as negative control. DNA was isolated using ZYMO ChIP DNA concentrator kit (D4014, ZYMO research). Libraries were generated using NEBNext Ultra II DNA Library Prep Kit for Illumina (E7645S, New England Biolabs) and subjected to 150 bp paired-end sequencing on an Illumina NovaSeq 6000.

ChIP-seq, ChIRP-seq data analysis

Raw sequencing reads were cleaned to remove adaptor sequences with cutadapt (version 1.18) (1). Trimmed reads were aligned to mouse reference genome (mm10) using Bowtie2 (version 2.5.2) (5) with parameters "--local --very-sensitive -p 20 -n 1". Alignment sam files were converted to bam file using samtools (11). Bam files were sorted and filtered to keep only unique reads using sambamba (version 0.6.9) (12). Peak calling was performed using MACS2 (version 2.2.6) (7). Treatment bedgraph is converted to bigWig file using bedGraphToBigWig for visualization. ChIP/ChIRP-seq tracks were viewed using the Integrated Genomic Viewer (version 2.8.13) (8). Differential peaks were indentified using DiffBind (version 2.10.0) (9) and followed ChIPseeker (version 1.28.3) (10) for annotation. All genomic datasets were deposited in the NCBI GEO under accession number (GEO: GSE269258).

Cleavage Under Targets and Release Using Nuclease (CUT&RUN) assay

CUT&RUN assays were performed using CUTANA™ ChIC/CUT&RUN Kit (14-1048, EpiCypher).

Briefly, $5x10^5$ cells were collected and washed in washing buffer (20mM HEPES pH7.5, 150mM NaCl, 0.5mM Spermidine, 1X EDTA-free protease inhibitor). Activated ConA beads were added in washed cells and incubated for 10 min at room temperature. ConA beads

bounded cells were incubated with 0.5ug of Anti-H3K4me3 or IgG (included in CUTANA™ ChIC/CUT&RUN Kit) for overnight at 4°C. The bead bound cells were washed with Digitonin buffer (20mM HEPES pH7.5, 150mM NaCl, 0.5mM Spermidine, 1X EDTA-free protease inhibitor, 0.01% digitonin), digested with 2.5ul of pAG-MNase for 10 min at room temperature. DNA was purified using CUTANA™ DNA Purification Kit. Libraries were generated using NEBNext Ultra II DNA Library Prep Kit for Illumina (E7645S, New England Biolabs) and subjected to 150 bp paired-end sequencing on an Illumina NovaSeq 6000.

Raw sequencing reads were cleaned to remove adaptor sequences with cutadapt (version 1.18) (1). Trimmed reads were aligned to mouse reference genome (mm10) using Bowtie2 (version 2.5.2) (5) with parameters "--local --very-sensitive --no-mixed --no-discordant --phred33 -I 10 -X 700". Alignment sam files were filtered with Picard MarkDuplicates (http://broadinstitute.github.io/picard, version 2.26.10) and converted to bam file using samtools (11). Bam files were converted to bigwig file using deepTools bamCoverage (version 3.1.3) (13). All genomic datasets were deposited in the NCBI GEO under accession number (GEO: GSE269258).

High throughput Chromosome Conformation Capture (Hi-C) Assay

Briefly, $1x10^7$ cells were cross-linked in 1 % formaldehyde (BP531, Fisher Bioregents) at room temperature for 10 min and quenched with 125 mM glycine solution. The cross-linked cell pellet was treated with lysis buffer and incubated at 4°C for 15 min. The extracted DNA from lysed cells was digested with restriction enzyme cocktail including in Arima-HiC Kit at 37°C for 30 min followed by at 65°C for 20 min. The 5'-overhangs of the digested DNA were filled ends and labeled with a biotinylated nucleotide, and ligated. 1µg of purified DNA per sample was sheared by sonication (Bioruptor, Diagenode) with default parameters (30 seconds ON, 30 seconds OFF pulse intervals) to obtain average 400bp length. Fragmented DNA was size-selected to have a size distribution between 200-600 bp. After enrichment of biotinylated DNA,

library was constructed using KAPA Hyper Prep Kit (KK8504, Roche) and submitted to paired-end sequencing of 150 bp length on an Illumina Novaseq.

Chromosome conformation capture combined with chromatin immunoprecipitation (HiChIP) and sequencing assay

HiChIP assay was performed in 961C and 961C *HoxBlinc* KO cells as previously described with a minor modification (14). Briefly, $2x10^7$ cells were crosslinked with 2% formaldehyde (BP531, Fisher Bioregents) for 10 min and subsequently quenched with 125mM glycine. Chromatin was digested using DpnII restriction enzyme (R0543M, New England Biolabs), followed by biotin incorporation with Biotin-14-dATP (19524016, Invitrogen). DNA was then ligated with T4 ligase (M0202M, New England Biolabs) and sonicated using Bioruptor (4 cycles (30-sec on, 30-sec off) set at position “high”). Sheared chromatin was diluted with ChIP dilution buffer (15mM Tris pH 7.5, 150mM NaCl, 1.2mM EDTA, 0.01% SDS, 1.1% Triton X-100), and cleaned with IgG (2729, Cell Signaling Technology) and then incubated with anti-CTCF antibody (91285, Active Motif) at 4°C overnight. Chromatin-antibody complexes were captured by Protein-A magnetic beads (10002D, Thermo Fisher Scientific) at 4°C for 4 hours, and subsequently washed with Low Salt Wash Buffer, High Salt Wash Buffer, LiCl Wash Buffer and eluted with elution buffer. After that, chromatin DNA was purified with ChIP DNA Clean & Concentrator (D5205, ZYMO) and quantified using Qubit High Sensitivity Assay kit (Q32851, Thermo Fisher Scientific). 50-150ng of DNA was used for capture with Dynabeads MyOne Streptavidin C-1 (65001, Thermo Fisher Scientific). Captured DNA was treated with Tn5 enzyme (20034197, Illumina). A first PCR in a final volume of 50µl with 5 cycles was performed (72°C for 5 min, 98°C for 1 min, then 5 cycles at 98°C for 15 s, 63°C for 30 s, and 72°C for 1 min). To determine the additional PCR cycles for optimal library preparation, the 30 cycles of qPCR reaction was performed using 5ul of first PCR product. The optimal number of additional cycles for each library was determined by setting a threshold of one-

third of the maximum fluorescent intensity. The 45µl left from the first PCR was further amplified using additional PCR cycles as determined above. Around 300 bp DNA fragments were selected using AMPure XP beads (A63880, Beckman Coulter). HiChIP libraries were quantified and analyzed using Qubit dsDNA HS Assay Kit (Q32851, Thermo Fisher Scientific). The libraries were sequenced paired-end at 150 bp length on Illumina NovaSeq 6000 platform.

Hi-C and HiChIP sequencing data analysis

Raw sequencing reads were cleaned to remove adaptor sequences with cutadapt (version 1.18) (1) and trimmed the end of enzymatic sequences with homerTools (homerTools trim -3 GATC -mis 0 -matchStart 20 -min 20) from Homer (version 4.10) (15). Trimmed reads were aligned to mouse reference genome (mm10) using Bowtie2 (version 2.5.2) (5) with parameters "--local --very-sensitive -p 20 -n 1". Aligned sam files were used to make a tag directory by makeTagDirectory package from Homer (15) with "-tbp 1" parameter to remove PCR duplicates. After filtering uninformative reads using makeTagDirectory package with different parameters "-genome mm10 -removePEbg -restrictionSite GATC -both -removeSelfLigation -removeSpikes 10000 5", the filtered tag directory file was converted to .hic files using tagDir2hicFile.pl. These interaction matrices for Hi-C heatmap were visualized by Juicebox (version 2.13.07) (16) using KR normalization with "Balanced" option. All genomic datasets were deposited in the NCBI GEO under accession number (GEO: GSE269258).

Single cell RNA-seq

Briefly, $1x10^4$ LK cells were loaded in each channel. Reverse transcription and library preparation were performed on C1000 Touch Thermal cycler with 96-Deep Well Reaction Module (Bio-Rad). Differentially barcoded libraries were diluted to 4nM and pooled for

sequencing with the NovaSeq 6000 Sequencing System (Illumina). Samples were sequenced with an average of 40,000 reads per cell.

Single cell RNA-seq analysis

Briefly, pooled samples were demultiplexed with cellranger mkfastq program (10x Genomics), and demultiplexed FASTQ files were then performed the alignment with reference mm10 genome, filtering, barcode counting, and unique molecular identifier (UMI) counting with cellranger count under the default parameters (10x Genomics). QC filtering was performed, and low complexity cell barcodes with number of genes detected were filtered out using the following parameters: percentage of reads mapping to the mitochondrial chromosome < 10%; UMI counts per cell > 500; and number of detected genes per cell (normalized counts > = 1) > 250. After filtering, we obtained a total of 9271 cells from WT LK group, a total of 9506 cells from *HoxBlinc*-Tg LK group. Additionally, 11,314 ± 49.23 (mean ± SD) UMIs per cell and an average of 2862 ± 45.87 (mean ± SD) genes per cell were detected. Then, data normalization, integration, clustering and dimensionality reduction T-distributed stochastic neighbor embedding (tSNE) or uniform manifold approximation and projection (UMAP) was performed using 'Seurat' package (17) in R language. Once data were successfully integrated, principal component analysis (PCA) by running the RunPCA function were performed with default parameters, and then by running the FindNeighbors function with reduction = "pca" and dims = 1:30, followed by running FindClusters function. For visualization, a Uniform Manifold Approximation and Projection (UMAP) cell embedding was generated using the RunUMAP function with the following parameters: reduction = "umap", dims = 1:30. Defining the clusters was manually assigned and curated on the basis of expressed genes previously reported (18, 19). Additionally, differentially expressed genes were calculated using Bonferroni corrected Wilcoxon Sum-Rank Test as implemented in FindAllMarkers function (default parameters) of the 'Seurat' package with adjusted p values < 0.05. FeaturePlot, CoveragePlot and DotPlot were performed to show the specific gene expression in each cell and cluster using 'Seurat' package. Average expression

level of the specific genes in different clusters was calculated with AverageExpression function in ‘Seurat’ package. We performed clustering of cells using louvain algorithm in SCANPY (20). In addition, we carried out PAGA analysis using sc.tl.paga function to explain the connectedness of the clusters (21). Trajectory inference analysis was performed with plot_cell_trajectory program in ‘Monocle’ package (22) and PAGA in Python (21). All genomic datasets were deposited in the NCBI GEO under accession number (GEO: GSE269258).

References:

1. Martin M. Cutadapt Removes Adapter Sequences From High-Throughput Sequencing Reads. EMBnet Journal2011. p. 10-2.
2. Kim D, et al. Graph-based genome alignment and genotyping with HISAT2 and HISAT-genotype. Nature Biotechnology. 2019;37(8):907-+.
3. Liao Y, et al. featureCounts: an efficient general purpose program for assigning sequence reads to genomic features. Bioinformatics. 2014;30(7):923-30.
4. Love MI, et al. Moderated estimation of fold change and dispersion for RNA-seq data with DESeq2. Genome Biology. 2014;15(12).
5. Langmead B and Salzberg SL. Fast gapped-read alignment with Bowtie 2. Nature Methods. 2012;9(4):357-U54.
6. Barnett DW, et al. BamTools: a C++ API and toolkit for analyzing and managing BAM files. Bioinformatics. 2011;27(12):1691-2.
7. Zhang Y, et al. Model-based Analysis of ChIP-Seq (MACS). Genome Biology. 2008;9(9).
8. Thorvaldsdottir H,et al. Integrative Genomics Viewer (IGV): high-performance genomics data visualization and exploration. Briefings in bioinformatics. 2013;14(2):178-92.
9. Ross-Innes CS, et al. Differential oestrogen receptor binding is associated with clinical outcome in breast cancer. Nature. 2012;481(7381):389-U177.
10. Yu GC, et al. ChIPseeker: an R/Bioconductor package for ChIP peak annotation, comparison and visualization. Bioinformatics. 2015;31(14):2382-3.
11. Li H, et al. The Sequence Alignment/Map format and SAMtools. Bioinformatics. 2009;25(16):2078-9.
12. Tarasov A, et al. Sambamba: fast processing of NGS alignment formats. Bioinformatics. 2015;31(12):2032-4. Epub 20150219.
13. Ramírez F, et al. deepTools: a flexible platform for exploring deep-sequencing data. Nucleic Acids Res. 2014;42(Web Server issue):W187-91.
14. Mumbach MR, et al. HiChIP: efficient and sensitive analysis of protein-directed genome architecture. Nat Methods. 2016;13(11):919-22.
15. Heinz S, et al. Simple Combinations of Lineage-Determining Transcription Factors Prime *cis*-Regulatory Elements Required for Macrophage and B Cell Identities. Molecular Cell. 2010;38(4):576-89.
16. Durand NC, et al. Juicebox Provides a Visualization System for Hi-C Contact Maps with Unlimited Zoom. Cell Systems. 2016;3(1):99-101.
17. Butler A, et al. Integrating single-cell transcriptomic data across different conditions, technologies, and species. Nat Biotechnol. 2018;36(5):411-20.
18. Giladi A, et al. Single-cell characterization of haematopoietic progenitors and their trajectories in homeostasis and perturbed haematopoiesis. Nat Cell Biol. 2018;20(7):836-46.
19. Paul F, et al. Transcriptional Heterogeneity and Lineage Commitment in Myeloid Progenitors. Cell. 2015;163(7):1663-77.
20. Wolf FA, et al. SCANPY: large-scale single-cell gene expression data analysis. Genome Biol. 2018;19(1):15.
21. Wolf FA, et al. PAGA: graph abstraction reconciles clustering with trajectory inference through a topology preserving map of single cells. Genome Biol. 2019;20(1):59. Epub 20190319.
22. Trapnell C, et al. The dynamics and regulators of cell fate decisions are revealed by pseudotemporal ordering of single cells. Nat Biotechnol. 2014;32(4):381-6.

Supplemental Table 1. Antibodies used in flowcytometry

Antibodies	SOURCE	IDENTIFIER
Anti-Human CD45 antibody, mouse monoclonal, APC-conjugated	BD Biosciences	Cat#555485, Clone: HI30, RRID:AB_398600
Anti-mouse CD45.1 antibody, mouse monoclonal, APC-conjugated	Invitrogen	Cat#17-0453-82, Clone: A20 RRID:AB_469398
Anti-mouse CD45.2 antibody, mouse monoclonal, PE-conjugated	Invitrogen	Cat#12-0454-82, Clone:104, RRID:AB_465678
Anti-Mouse CD45R/B220, rat monoclonal, APC-conjugated	BD Pharmingen	Cat#553092, Clone:RA3-6B2, RRID: AB_398531
Anti-Mouse CD43, rat monoclonal, BV421-conjugated	BD OptiBuild	Cat# 752957, Clone: S7, RRID: N/A
Anti-Mouse IgM, rat monoclonal, APC-Cy7-conjugated	Biolegend	Cat# 406516, Clone: RMM-1, RRID: AB_10660305
Anti-Mouse CD117, rat monoclonal, APC-conjugated	BD Pharmingen	Cat# 553356, Clone:2B8, RRID: AB_398536
Anti-Mouse CD135, rat monoclonal, BV421-conjugated	BD Horizon	Cat# 562898, Clone: A2F10.1, RRID: AB_2737876
Mouse Lineage Antibody Cocktail FITC	Biolegend	Cat# 133302; Clone: 145-2C11, RB6-8C5, M1/70, RA3-6B2, Ter-119, RRID: AB_10697030
Anti-Mouse CD117, rat monoclonal, PE-conjugated	BD Pharmingen	Cat# 553355; Clone: 2B8, RRID: AB_394806
Anti-Mouse Ly-6A/E (Sca1), rat monoclonal, PE-Cy7-conjugated	BD Pharmingen	Cat# 558162, Clone: D7, RRID: AB_647253
Anti-Mouse Ly-6D, rat monoclonal, PE-conjugated	BD Pharmingen	Cat# 561149, Clone :49-H4, RRID: AB_10563763
Anti-Mouse CD127, rat monoclonal, BV605-conjugated	BD Horizon	Cat# 569295, Clone: A7R34, RRID: N/A
Anti-Mouse CD19, rat monoclonal, Alexa Fluor® 700-conjugated	BD Pharmingen	Cat# 557958, Clone: 1D3, RRID: AB_396958

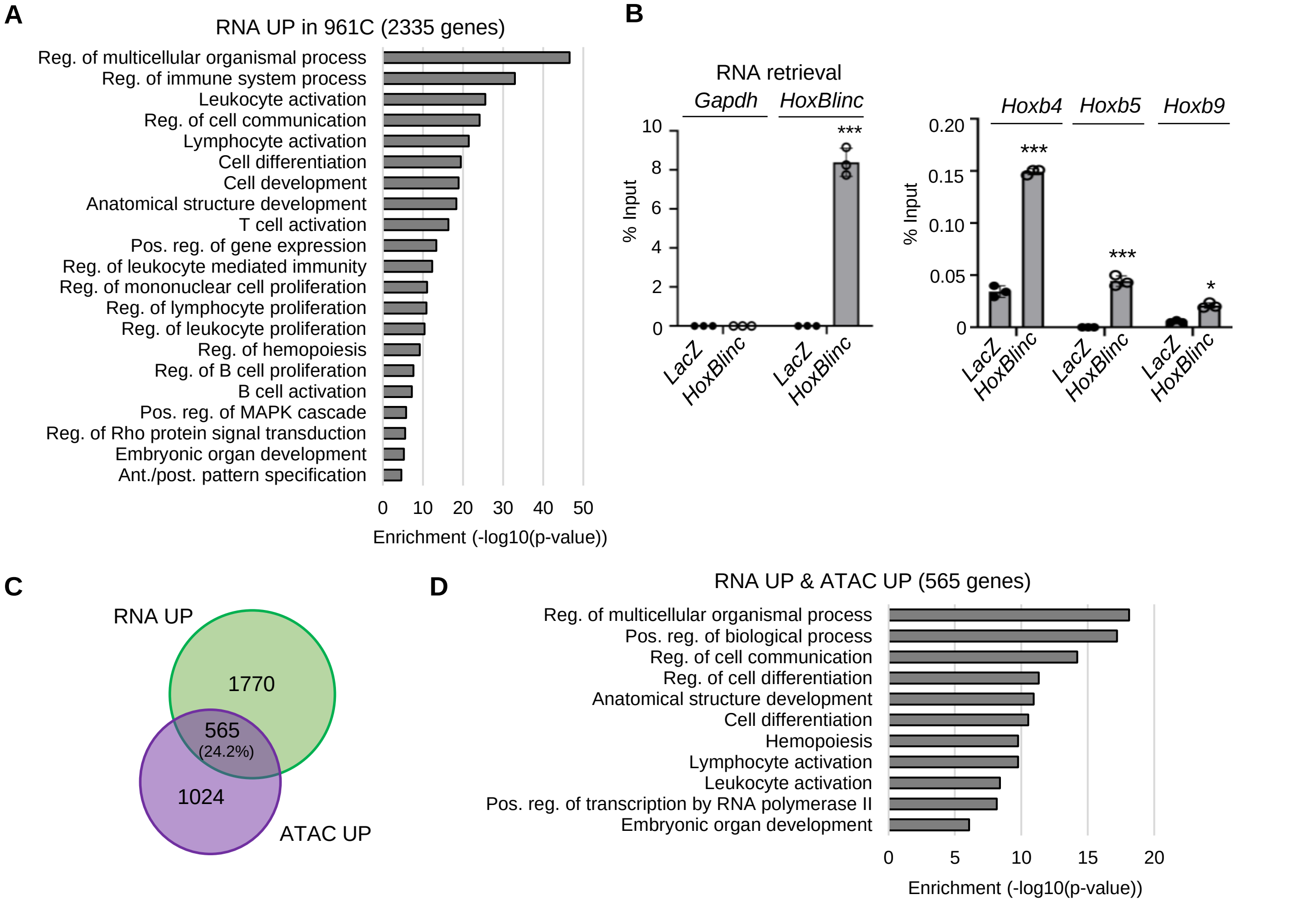

Supplementary Figure 1. Related to Figure 1; *HoxBlinc* lncRNA is highly activated and binds to Hoxa/b in NUP98-PHF23 driven leukemia. (**A**) Gene ontology analysis of upregulated RNA in 961C compared with BaF3. (**B**) (Left) *HoxBlinc* RNA retrieval precipitated with *HoxBlinc* biotinylated probes during ChIRP-assay by RT-qPCR. (Right) *HoxBlinc* ChIRP-qPCR at Hoxb4/Hoxb5/Hoxb9 locus. Data was presented as mean ± SD; $***p \leq 0.001$, $**p \leq 0.01$, $*p \leq 0.05$ by two-tailed unpaired *t* test. (**C**) Overlapping analysis of upregulated gene and upregulated ATAC peaks in 961C compared with BaF3. (**D**) Gene ontology analysis of overlapping 565 genes.

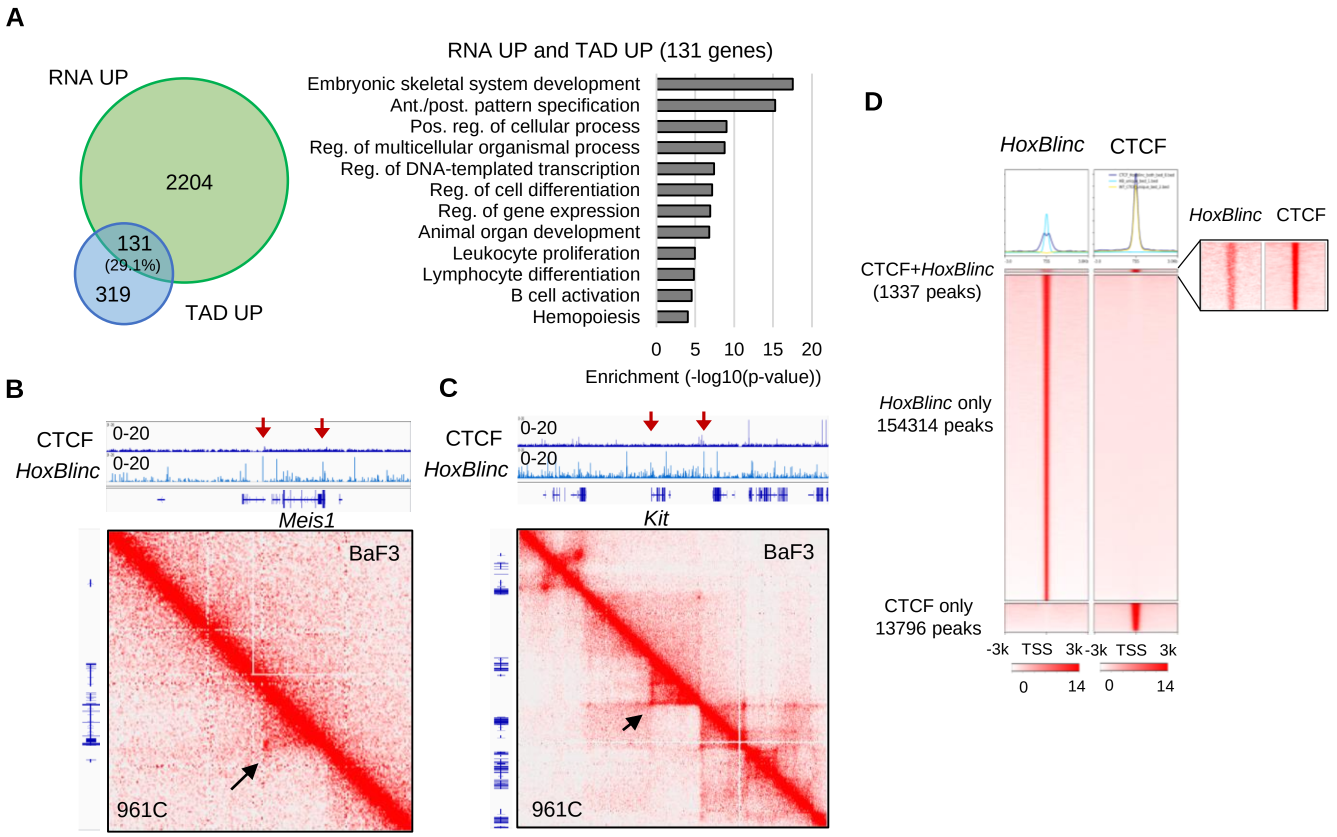

Supplementary Figure 2. Related to Figure 2; *HoxBlinc* defines chromatin boundaries of Hoxa/b TADs in NUP98-PHF23 driven leukemia. (A) (Left) Overlapping analysis of upregulated RNA and genes encompassed in upregulated TADs in 961C, (right) Gene ontology analysis of overlapped 131 genes. (B) HiC-seq track with CTCF ChIP and *HoxBlinc* ChIRP at Meis1 locus (Chr11:18300000-19300000) visualized by Juicebox and IGV. (C) HiC-seq track with CTCF ChIP and *HoxBlinc* ChIRP at Kit locus (Chr5:74800000-76600000) visualized by Juicebox and IGV. (D) Heatmaps of *HoxBlinc* binding and CTCF binding in 961C.

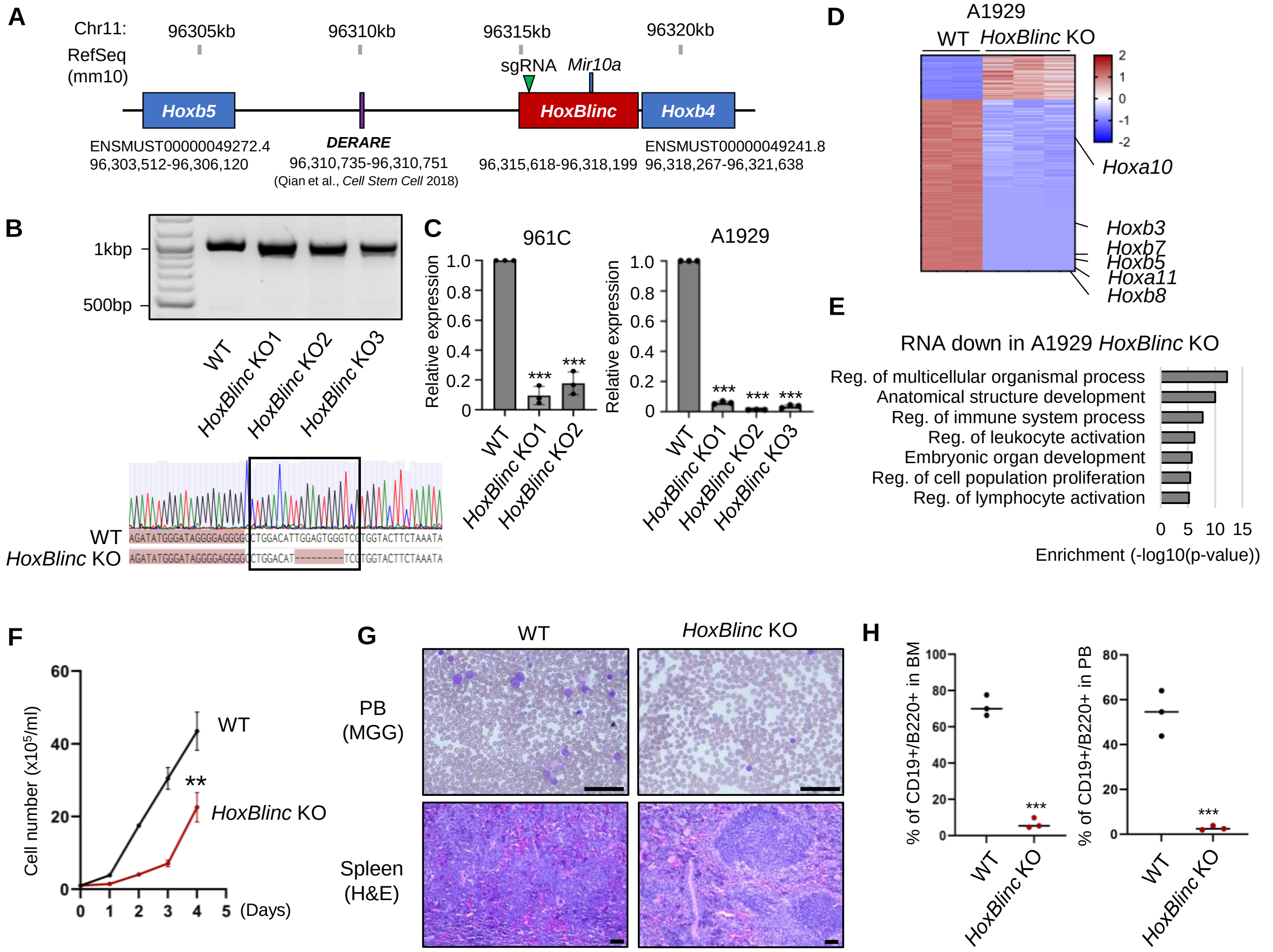

A
Chr11:
RefSeq (mm10)
96305kb
96310kb
96315kb
96320kb
sgRNA
Mir10a
Hoxb5
HoxBlinc
Hoxb4
DERARE
ENSMUST00000049272.4
96,303,512-96,306,120
96,310,735-96,310,751
(Qian et al., Cell Stem Cell 2018)
96,315,618-96,318,199
ENSMUST00000049241.8
96,318,267-96,321,638
B
1kbp
500bp
WT
HoxBlinc KO1
HoxBlinc KO2
HoxBlinc KO3
WT
HoxBlinc KO
C
961C
A1929
Relative expression
WT
HoxBlinc KO1
HoxBlinc KO2
HoxBlinc KO3
D
A1929
WT
HoxBlinc KO
Hoxa10
Hoxb3
Hoxb7
Hoxb5
Hoxa11
Hoxb8
E
RNA down in A1929 HoxBlinc KO
Reg. of multicellular organismal process
Anatomical structure development
Reg. of immune system process
Reg. of leukocyte activation
Embryonic organ development
Reg. of cell population proliferation
Reg. of lymphocyte activation
Enrichment (-log10(p-value))
F
Cell number (x10⁵/ml)
WT
HoxBlinc KO
(Days)
G
WT
HoxBlinc KO
PB (MGG)
Spleen (H&E)
H
% of CD19+/B220+ in BM
% of CD19+/B220+ in PB
WT
HoxBlinc KO

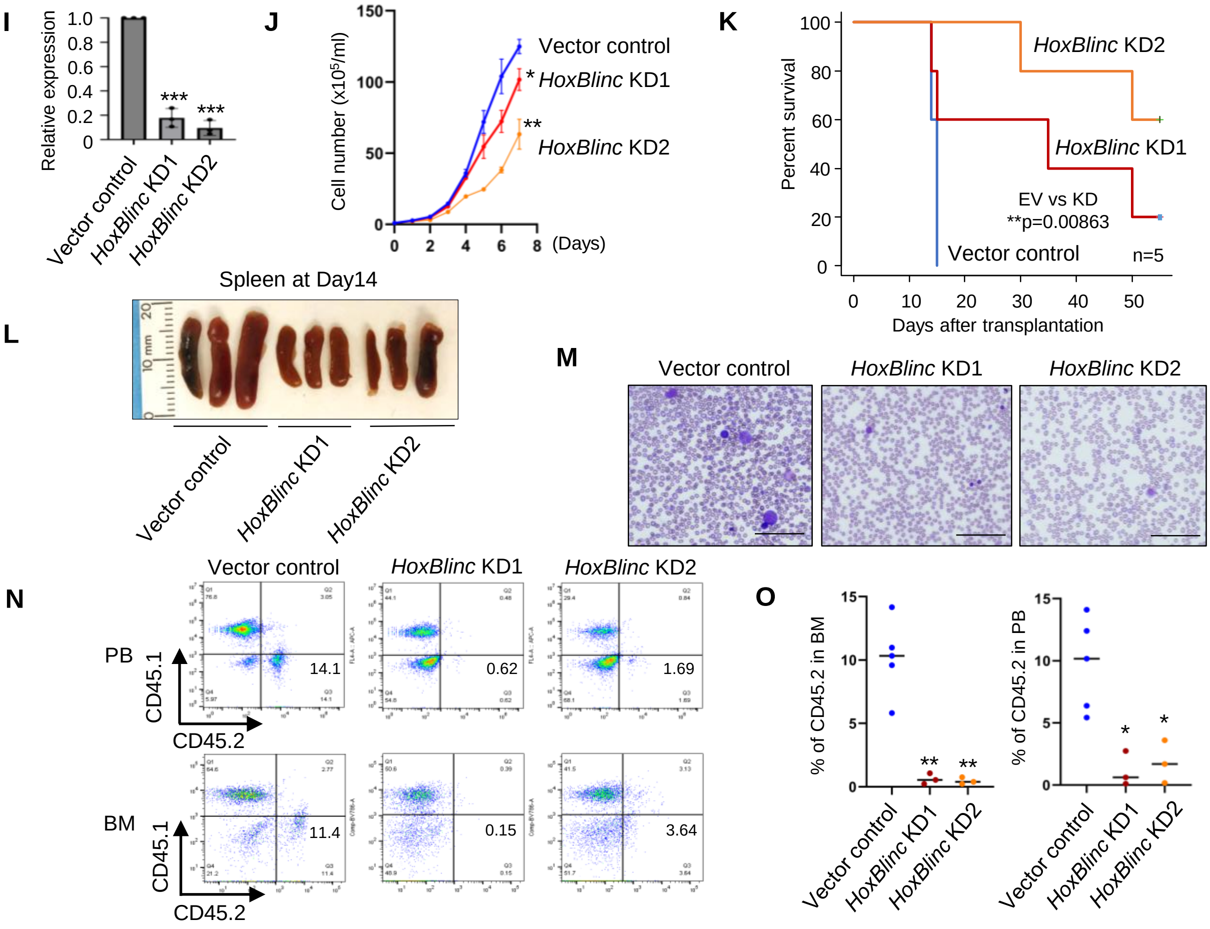

Supplementary Figure 3. Related to Figure 3; *HoxBlinc* lncRNA is essential for NUP98-PHF23 driven homeotic leukemic gene signature and leukemogenesis. (**A**) Gene locus of Hoxb4-Hoxb5 (mm10, Chr11:96000000-96330000). (**B**) Genotyping of *HoxBlinc* KO clone by PCR and sanger sequencing. Black box showed sgRNA sequence. (**C**) Relative expression of *HoxBlinc* gene of WT/*HoxBlinc* KO in 961C or A1929 by RT-qPCR. Data was presented as mean ± SD; ***p ≤ 0.001, **p ≤ 0.01, *p ≤ 0.05 by ANOVA following Bonferroni post hoc test. (**D**) Heatmap of differentially expressed genes in A1929 WT and *HoxBlinc* KO. (**E**) Gene ontology analysis of downregulated gene in A1929 *HoxBlinc* KO. (**F**) Cell proliferation curve for 4 days in A1292 WT/*HoxBlinc* KO. Data was presented as mean ± SD; ***p ≤ 0.001, **p ≤ 0.01, *p ≤ 0.05 by two-tailed unpaired *t* test. (**G**) (Top) May-Grunwald Giemsa staining of PB smear from A1929 WT/*HoxBlinc* KO transplanted mice at Day23, (Bottom) H&E staining of spleen from A1929 WT/*HoxBlinc* KO transplanted mice at Day23. Scale bar: 50µm. (**H**) Bar graph of CD19+/B220+ population in BM/PB from A1929 WT/*HoxBlinc* KO transplanted mice at Day23; N=3 in each group, ***p ≤ 0.001, **p ≤ 0.01, *p ≤ 0.05 by two-tailed unpaired *t* test. (**I**) Relative expression of *HoxBlinc* gene in 961C WT/*HoxBlinc* KD by RT-qPCR. Data was presented as mean ± SD; ***p ≤ 0.001, **p ≤ 0.01, *p ≤ 0.05 by ANOVA following Bonferroni post hoc test. (**J**) Proliferation curve for 7 days in 961C vector control and *HoxBlinc* KD. Data was presented as mean ± SD; ***p ≤ 0.001, **p ≤ 0.01, *p ≤ 0.05 by ANOVA following Bonferroni post hoc test. (**K**) Survival curve of mice transplanted with 961C vector control or *HoxBlinc* KD; N=5 in each group; p=0.00863 by log-rank test. (**L**) Spleen from 961C vector control or *HoxBlinc* KD transplanted mice at day14 post transplantation. (**M**) May-Grunwald Giemsa staining of PB smear from 961C vector control or *HoxBlinc* KD transplanted mice at day14 post transplantation. Scale bar: 50µm. (**N**) Flow cytometry of hCD45.2 in PB cells (Top) and BM cells (Bottom) from 961C vector control or *HoxBlinc* KD transplanted mice at day14 post transplantation. (**O**) Bar graph of hCD45.2 flowcytometry in BM or PB cells from 961C vector control or *HoxBlinc* KD transplanted mice at day14 post transplantation; N=5 for WT, N=3 for each clone of KD, ***p ≤ 0.001, **p ≤ 0.01, *p ≤ 0.05 by ANOVA following Bonferroni post hoc test.

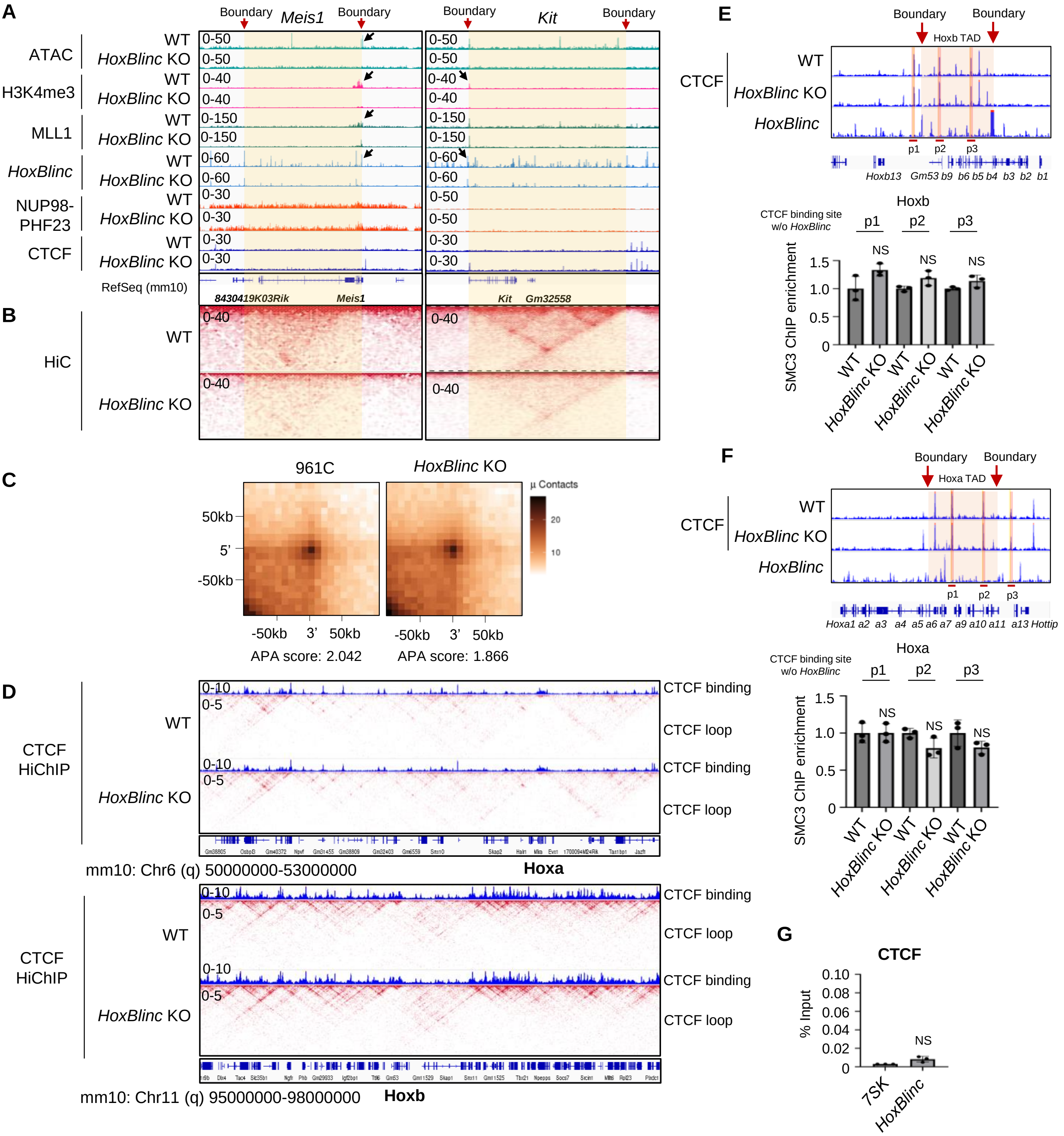
A
Boundary
Meis1
Boundary
Boundary
Kit
Boundary
ATAC
H3K4me3
MLL1
HoxBlinc
NUP98-PHF23
CTCF
WT
HoxBlinc KO
RefSeq (mm10)
8430419K03Rik
Meis1
Kit
Gm32558
B
HiC
WT
HoxBlinc KO
C
961C
HoxBlinc KO
µ Contacts
50kb
5'
-50kb
-50kb 3' 50kb
APA score: 2.042
APA score: 1.866
D
CTCF HiChIP
CTCF binding
CTCF loop
mm10: Chr6 (q) 50000000-53000000
Hoxa
mm10: Chr11 (q) 95000000-98000000
Hoxb
E
Boundary
Hoxb TAD
CTCF
HoxBlinc
p1 p2 p3
Hoxb13 Gm53 b9 b6 b5 b4 b3 b2 b1
Hoxb
CTCF binding site w/o HoxBlinc
SMC3 ChIP enrichment
NS
F
Boundary
Hoxa TAD
Hoxa1 a2 a3 a4 a5 a6 a7 a9 a10 a11 a13 Hottip
Hoxa
G
CTCF
% Input
7SK
HoxBlinc

H

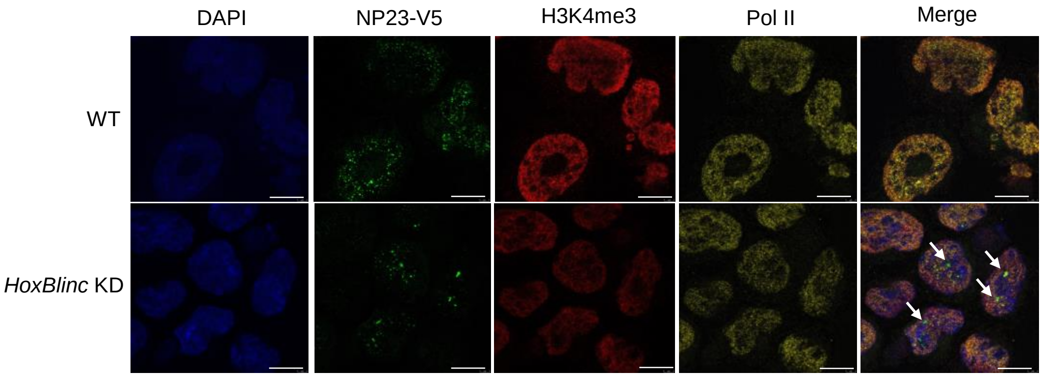

I

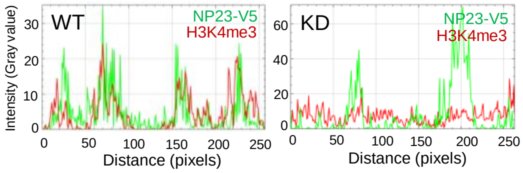

Supplementary Figure 4. Related to Figure 4; *HoxBlinc* loss Impairs aberrant TAD integrity and chromatin signature to drive NUP98-PHF23 driven leukemic transcription profile. (A) ATAC/H3K4me3 CUT&RUN/MLL1, NUP98-PHF23 and CTCF ChIP/*HoxBlinc* ChIRP track at *Meis1* locus (Chr11:18800000-19100000) and *Kit* locus (Chr5:75500000-75900000) in 961C WT/*HoxBlinc* KO visualized by IGV. **(B)** HiC track at *Meis1* locus (Chr11:18800000-19100000) and *Kit* locus (Chr5:75500000-75900000) in 961C WT/*HoxBlinc* KO visualized by WashU epigenome browser. **(C)** APA plot of 3003 CTCF-mediated loops in 961C WT/*HoxBlinc* **(D)** CTCF HiChIP track at Hoxb and Hoxa locus in 961C WT/*HoxBlinc* KO visualized by WashU epigenome browser. (**E**) SMC3 enrichment at non-*HoxBlinc* bounded CTCF binding site at Hoxb locus by SMC3 ChIP-qPCR. Data was presented as mean ± SD; No statistical significance by two-tailed unpaired *t* test. **(F)** SMC3 enrichment at non-*HoxBlinc* bounded CTCF binding site at Hoxa locus by SMC3 ChIP-qPCR. Data was presented as mean ± SD; No statistical significance by two-tailed unpaired *t* test. (**G**) RNA-IP qPCR precipitated with CTCF antibody in 961C. Data was presented as mean ± SD; No statistical significance by two-tailed unpaired *t* test. **(H)** Immunofluorescence staining with NUP98-PHF23-V5, H3K4me3 and Pol II in 961C WT/*HoxBlinc* KD. Scale bar: 10µm. **(I)** Colocalization analysis of NUP98-PHF23-V5 and H3K4me3 in 961C WT/*HoxBlinc* KD by ImageJ.

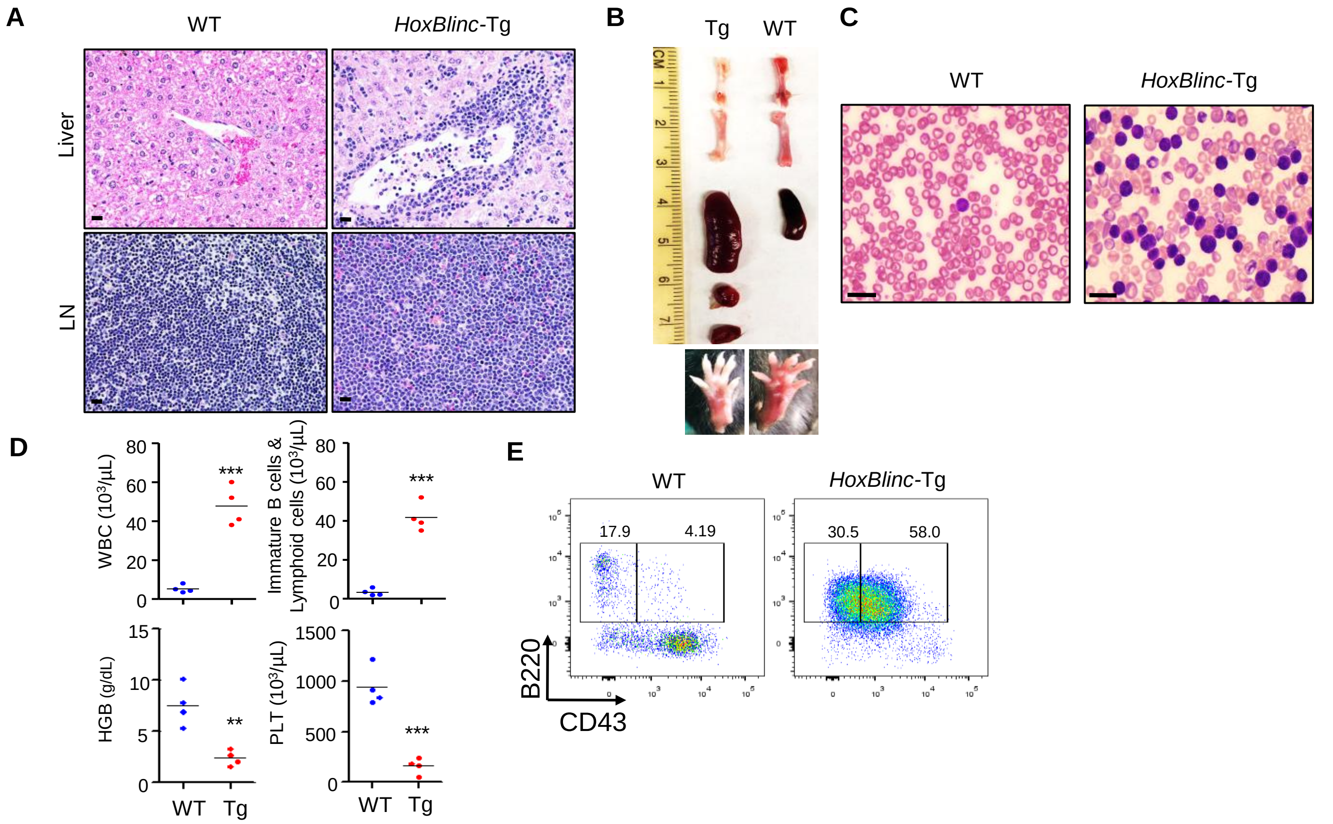

Supplementary Figure 5. Related to Figure 5; Transgenic expression of *HoxBlinc* in mouse hematopoietic compartment led to various leukemias. (A) H&E staining of liver/lymph node (LN) from representative WT and B-ALL *HoxBlinc*-Tg mice. Scale bar: 50µm. **(B)** Gross appearance of femur, spleens and lymph nodes as well as foot pats of representative recipients transplanted with WT or B-ALL *HoxBlinc*-Tg mice. **(C)** May Grunwald-Giemsa staining of PB smear from recipients receiving WT or B-ALL *HoxBlinc*-Tg spleen cells. Scale bar: 50µm. **(D)** PB counts from recipient mice transplanted with WT or B-ALL *HoxBlinc*-Tg cells; N=4, ***$p \leq 0.001$, **$p \leq 0.01$, *$p \leq 0.05$ by two-tailed unpaired *t* test. **(E)** Flow cytometry analysis of B-cell populations in the BM of recipients receiving WT or B-ALL *HoxBlinc*-Tg mice.

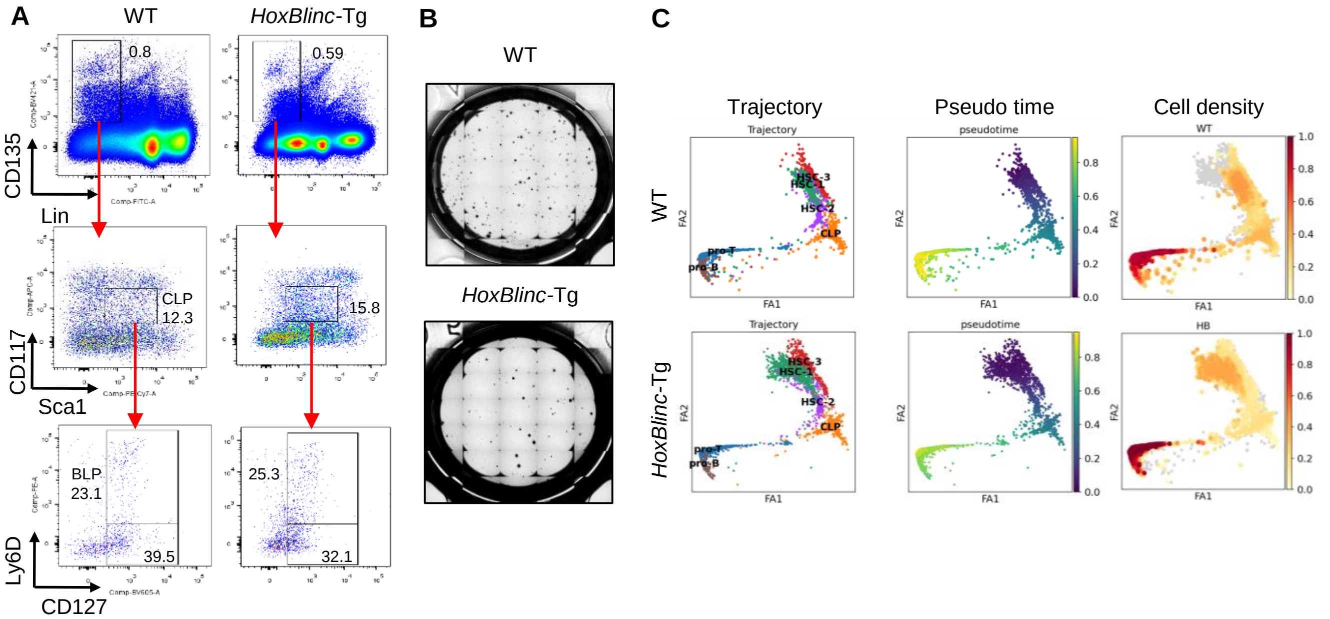

Supplementary Figure 6. Related to Figure 6; *HoxBlinc* overexpression impairs B cell development. (A) Flow cytometry gate strategy to identify BLP and CLP population in BM from WT/*HoxBlinc*-Tg mice. **(B)** Images of the B-cell colony assays of WT and *HoxBlinc*-Tg BM cells in the presence of IL-7. **(C)** Trajectory, pseudo time and cell density determined by single cell RNA-seq using WT/*HoxBlinc*-Tg LK cells.

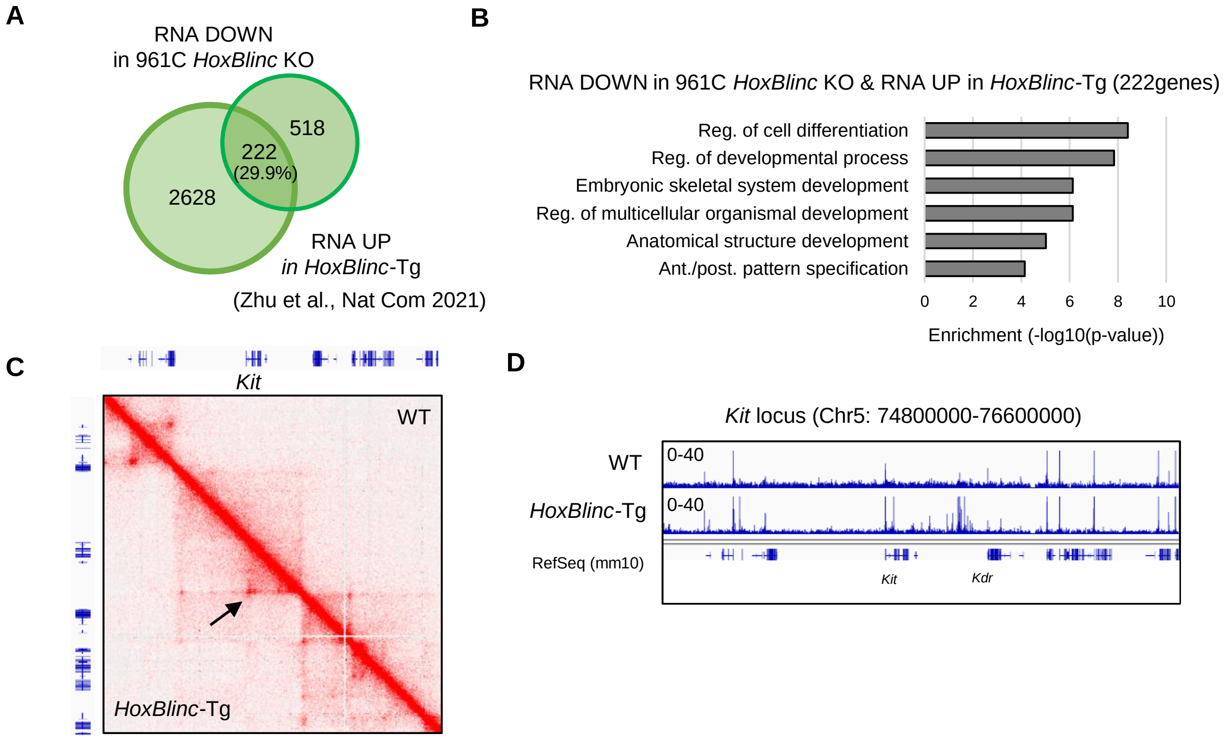

Supplementary Figure 7. Related to Figure 7; Transgenic expression of *HoxBlinc* activates leukemic stem like gene TADs and chromatin signature. (A) Overlapping analysis of downregulated RNA in 961C *HoxBlinc* KO compared with 961C WT and upregulated RNA in *HoxBlinc*-Tg compared with parental mice (Zhu et al. Nat Com 2021). **(B)** Gene ontology analysis of overlapped 222 genes. **(C)** HiC track at *Kit* locus (Chr5:75500000-75900000). Black arrow indicates upregulated TAD in *HoxBlinc*-Tg mice at *Kit* locus. **(D)** ATAC-seq track at *Kit* locus (Chr5:75500000-75900000).

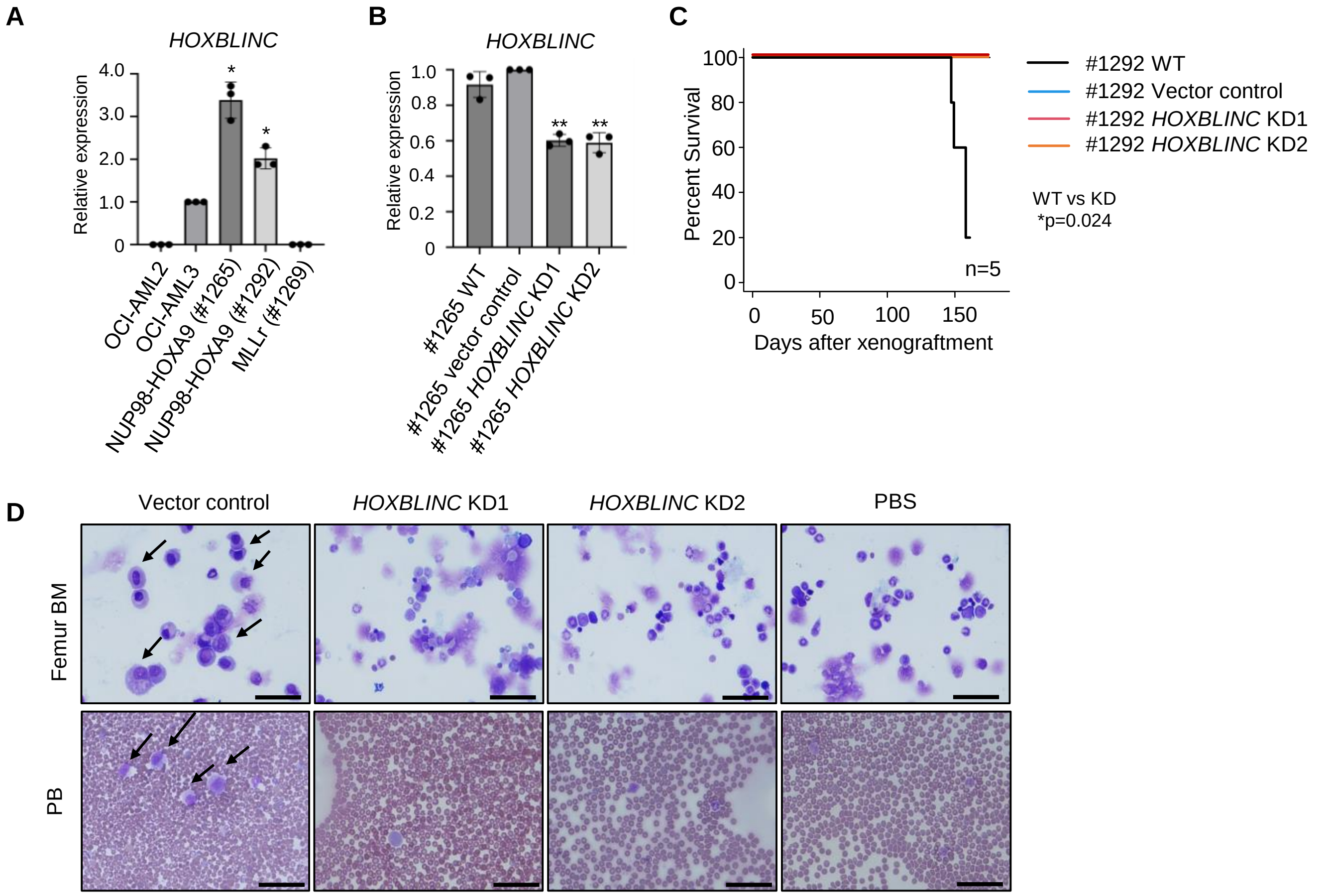

Supplementary Figure 8. Related to Figure 8; *HOXBLINC* lncRNA is also required for NUP98-HOXA9 driven homeotic gene transcription and leukemic transformation. (**A**) Relative expression of *HOXBLINC* in primary AML patient samples. Data was presented as mean ± SD; ***p ≤ 0.001, **p ≤ 0.01, *p ≤ 0.05 by ANOVA following Bonferroni post hoc test. (**B**) Relative expression of *HOXBLINC* in WT, vector control and *HOXBLINC* KD in NUP98-HOXA9 AML patient #1265. Data was presented as mean ± SD; ***p ≤ 0.001, **p ≤ 0.01, *p ≤ 0.05 by ANOVA following Bonferroni post hoc test. (**C**) Survival curve of mice xenografted with #1292 WT, vector control or *HOXBLINC* KD; N=5 in each group; p=0.024 WT vs KD by log-rank test following Bonferroni post hoc test. (**D**) MGG staining of Femur BM and PB smear from #1265 vector control or *HOXBLINC* KD, PBS injected NSGS mice at day143 post xenograftment. Scale bar: 50µm.
