# Supplementary figures and images for: HoxBlinc lncRNA reprograms CTCF-independent TADs to drive leukemic transcription and HSC dysregulation in NUP98-rearranged leukemia
Source: J Clin Invest. 2025 Jan 30;135(7):e184743. doi: 10.1172/JCI184743 (PMC11957699; doi:10.1172/JCI184743)

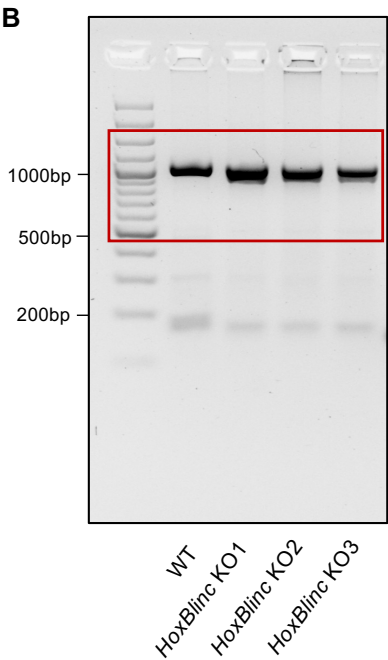

Supplement: Unedited blot and gel images [file jci-135-184743-s234.pdf]
